# Assessment of Heavy Metal Accumulation in Soils and Dominant Agricultural Crops in an Industrial Environment of Ridder, East Kazakhstan Region

**DOI:** 10.3390/plants15060983

**Published:** 2026-03-23

**Authors:** Dias Daurov, Kabyl Zhambakin, Ainash Daurova, Zagipa Sapakhova, Iskander Isgandarov, Raushan Ramazanova, Moldir Zhumagulova, Aidar Sumbembayev, Zhanar Abilda, Maxat Toishimanov, Rakhim Kanat, Malika Shamekova

**Affiliations:** 1 Laboratory of Breeding and Biotechnology, Institute of Plant Biology and Biotechnology, Timiryazev 45, Almaty 050040, Kazakhstan; 2 Faculty of Agrobiology, Kazakh National Agrarian Research University, Almaty 050010, Kazakhstan; 3 Tanir Research Laboratory, 75B Al-Farabi Avenue, Almaty 050060, Kazakhstan; 4 U.U. Uspanov Kazakh Research Institute of Soil Science and Agrochemistry, Al-Farabi Ave., 75 B, Almaty 050060, Kazakhstan; 5 Altai Botanical Garden, Ridder 071300, Kazakhstan

**Keywords:** heavy metals, soil contamination, ecological risk, phytostabilization, industrial impact, Ridder, plant communities

## Abstract

Mining and metallurgical activities are among the main sources of heavy metal (HM) contamination of terrestrial ecosystems and the creation of persistent technogenic pollution hotspots. This study aimed to provide a comprehensive assessment of the accumulation of zinc (Zn), cooper (Cu), cadmium (Cd) and lead (Pb) in soils and vegetation under conditions of long-term industrial impact in Ridder, East Kazakhstan Region. A total of 52 soil samples were collected from 0–5 cm and 5–20 cm depths at 26 sites, and 44 species of natural vegetation, as well as three dominant agricultural crops, were examined. Soil concentrations of Zn (4415 mg·kg^−1^), Cu (1177 mg·kg^−1^), Cd (179 mg·kg^−1^), and Pb (1996 mg·kg^−1^) were classified as extremely high. Cadmium contributed most to the potential ecological risk (Cd > Pb > Zn > Cu). The industrial zone’s vegetation cover was predominantly formed by stress-tolerant and ruderal species, including *Artemisia vulgaris*, *Calamagrostis epigeios*, *Bunias orientalis*, *Dactylis glomerata*, *Convolvulus arvensis*, and *Urtica dioica*. The agricultural crops (*Helianthus annuus*, *Avena sativa*, and *Triticum aestivum*) mainly accumulated HMs in their root systems, with limited translocation to their aboveground organs (TF < 1). This indicates the predominance of phytostabilisation mechanisms, and highlights the potential of locally adapted plants for managing contaminated areas.

## 1. Introduction

Heavy metal contamination has become a global environmental concern due to the toxicity of these elements and their ability to accumulate in soils over time [1]. As a rule, heavy metal pollution is primarily associated with anthropogenic activities such as mining, the application of mineral fertilizers, vehicular emissions, and various industrial operations. Industrial activity is widely regarded as the principal source of heavy metal contamination [2]. A representative example of such technogenic impact is the city of Ridder in the East Kazakhstan Region, an industrial center characterized by intensive mining and metallurgical activities [3,4]. The extraction and beneficiation of polymetallic ores, mainly lead–zinc deposits, have resulted in persistent anthropogenic pressure on environmental components. Prolonged operation of mines, processing plants, and metallurgical facilities has led to substantial alterations in soil cover and vegetation.

A direct outcome of such prolonged industrial pressure is the formation of a specific type of anthropogenic contamination characterized by the predominant accumulation of heavy metals [5]. These elements are distinguished by high persistence and long-term retention in the soil environment. Therefore, the soils of Ridder and adjacent areas act as the main sink for technogenic emissions. Moreover, the uneven distribution of pollutants across the territory and in the soil profile reflects not only the intensity of industrial impact, but also the influence of natural factors, including particle size distribution, organic matter content and acid–base conditions [6,7]. The combined effect of these parameters creates specific edaphic conditions that govern metal migration, bioavailability, and the level of potential ecological risk, particularly in the surface soil layer [8].

Against this background, heavy metals such as Zn, Cu, Cd, and Pb are of particular ecological concern owing to their effects on biota. Zinc and copper are biogenic elements. However, at elevated concentrations they exhibit pronounced phytotoxic properties, whereas cadmium and lead are among the most hazardous pollutants owing to their high toxicity and lack of biological function [9,10,11]. The behavior of these elements in technogenically transformed landscapes is determined by the complex interaction of soil physicochemical properties, the sorption capacity of fine fractions, and the role of organic matter, which together control the processes of retention, vertical migration, and accumulation of metals within the soil profile [12,13].

The state of vegetation cover represents a direct manifestation of these processes, as vegetation serves both as a sensitive indicator of soil contamination and as an active participant in heavy metal migration within the soil–plant system [14]. Under conditions of chronic industrial impact, phytocenosis formation is constrained by strong edaphic limitations, leading to the dominance of stress-tolerant, perennial, and ruderal species [15]. At the same time, agricultural crops cultivated on technogenically transformed lands are capable of accumulating heavy metals in their biomass [16]. In this regard, particular importance is attached to the analysis of species-specific accumulation patterns and the balance between metal retention in the root system and translocation to aboveground organs [17].

Despite numerous studies devoted to heavy metal contamination in mining regions, several important aspects remain insufficiently understood. Previous investigations have primarily focused on the assessment of total heavy metal concentrations and general pollution indices, whereas comparatively little attention has been paid to the integrated analysis of soil physicochemical properties, particle-size composition, ecological risk indicators, and plant accumulation processes within a unified soil–plant system [18]. In particular, the mechanisms governing the spatial distribution and vertical differentiation of heavy metals in soils subjected to long-term polymetallic mining in the Ridder industrial region remain insufficiently characterized.

Given the multifactorial nature of interactions between soil and biological components in contaminated ecosystems, their investigation requires the application of integrated approaches. The integration of data on soil physicochemical properties, contamination levels, and ecological risk indicators using modern multivariate statistical methods allows a deeper understanding of the mechanisms governing heavy metal distribution and biotic responses [19]. In particular, ordination techniques such as redundancy analysis (RDA) and non-metric multidimensional scaling (NMDS), combined with correlation analysis, facilitate the identification of key factors shaping spatial contamination patterns and the structural organization of plant communities along gradients of technogenic impact [20].

The conceptual framework of the present study is based on an integrated soil–plant system approach, in which industrial emissions act as the primary source of heavy metals, while soil physicochemical properties regulate their retention, mobility, and bioavailability [21]. These processes ultimately determine the patterns of metal accumulation in plants and the associated ecological risks within technogenically transformed landscapes.

The aim of the present study is to provide a comprehensive assessment of the distribution of Zn, Cu, Cd, and Pb in soils subjected to long-term industrial impact, using contamination and potential ecological risk indices, and to elucidate the role of soil physicochemical properties and particle-size composition in governing metal behavior. In addition, the structure of vegetation cover and the accumulation of heavy metals by dominant agricultural crops are examined, including analyses of bioconcentration and translocation factors. Such an integrated soil–plant approach provides a scientific basis for objective ecological risk assessment and the development of strategies for the sustainable management of technogenically transformed territories.

Based on this framework, we hypothesize that (1) the spatial distribution and vertical differentiation of Zn, Cu, Cd, and Pb in soils are largely controlled by soil physicochemical properties and particle-size composition, particularly the proportion of fine fractions and organic matter content, and (2) these soil characteristics significantly influence the bioaccumulation and translocation of heavy metals in plants growing under conditions of long-term technogenic impact.

## 2. Results

### 2.1. Soil

#### 2.1.1. Physicochemical Properties of Soils

**Figure 1:**
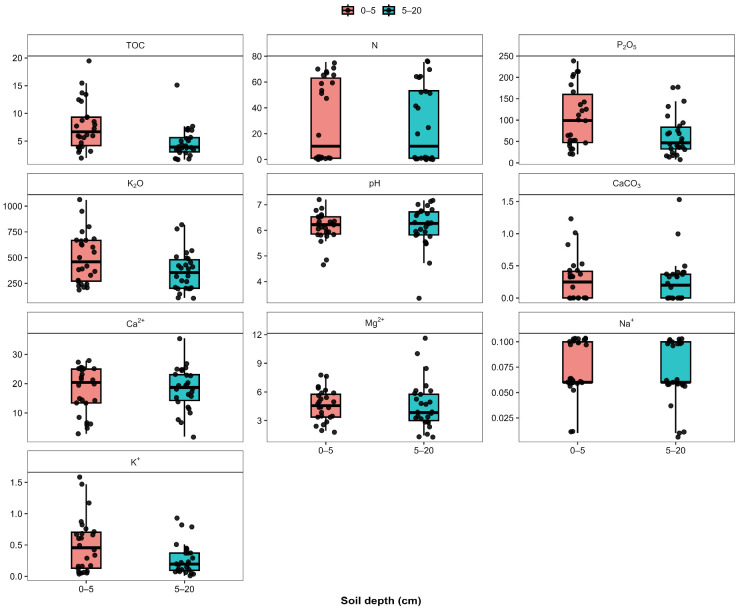
Boxplot distribution of selected soil agrochemical and physicochemical properties at two depths (0–5 and 5–20 cm). Boxes represent the interquartile range, the central line shows the median, whiskers indicate the data range, and points represent individual sampling sites.

The agrochemical and physicochemical properties of soils collected from 26 sampling sites were analyzed at two depths (0–5 and 5–20 cm). Overall, the results reveal considerable variability among the measured parameters as well as noticeable differences between the surface and subsurface soil horizons (Figure 1, Table S1).

Among the studied indicators, total organic carbon (TOC) showed one of the most pronounced vertical gradients. The surface layer (0–5 cm) was characterized by higher median values and greater variability compared with the 5–20 cm layer, indicating the preferential accumulation of organic carbon in the upper soil horizon. In contrast, the subsurface layer exhibited generally lower TOC concentrations and a narrower distribution range.

A similar vertical pattern was observed for available nitrogen, which showed higher concentrations and wider dispersion in the surface layer relative to the deeper horizon. When compared with TOC, nitrogen displayed a comparable depth-related trend, suggesting that both parameters follow a similar distribution pattern within the soil profile, with enrichment in the upper horizon.

In contrast to nitrogen, the distribution of available phosphorus (P_2_O_5_) was characterized by substantially greater variability across the sampling sites. While phosphorus concentrations were often higher in the surface layer, several samples exhibited comparable values in both horizons. Consequently, the vertical differentiation of phosphorus was less consistent than that observed for TOC and nitrogen.

A comparable tendency was observed for exchangeable potassium (K_2_O). Although potassium concentrations varied widely among samples, the difference between soil depths was generally less pronounced than for nitrogen and TOC. Median values in the two layers were relatively similar, indicating a more uniform distribution of potassium within the upper soil profile.

When considering soil chemical conditions, pH values ranged from moderately acidic to near neutral and showed relatively small differences between the two depth intervals. Compared with nutrient parameters such as nitrogen and phosphorus, soil pH exhibited lower variability and largely overlapping distributions between depths, suggesting relatively stable acidity conditions within the upper soil profile.

The content of calcium carbonate (CaCO_3_) remained generally low across most samples. Occasional elevated values were observed, but these appeared as isolated cases rather than forming a systematic pattern. In comparison with nutrient elements, CaCO_3_ displayed a weaker depth-related trend and relatively limited variability.

The composition of exchangeable base cations further highlights differences among the measured chemical parameters. Among the analyzed cations, Ca^2+^ was clearly dominant and exhibited higher concentrations than the other exchangeable bases. Mg^2+^ also contributed substantially to the exchange complex, although its concentrations were generally lower than those of calcium. In contrast, Na^+^ and K^+^ represented only a minor proportion of the exchangeable cation pool and remained consistently low across all samples.

In general, comparison of the studied parameters indicates that TOC and available nitrogen showed the most distinct vertical differentiation, with higher concentrations in the surface layer. In contrast, phosphorus and potassium exhibited greater spatial variability but weaker depth-related differences, while pH, CaCO_3_, and exchangeable base cations displayed relatively stable distributions within the upper of the soil profile.

#### 2.1.2. Particle-Size Distribution and Soil Texture

**Figure 2:**
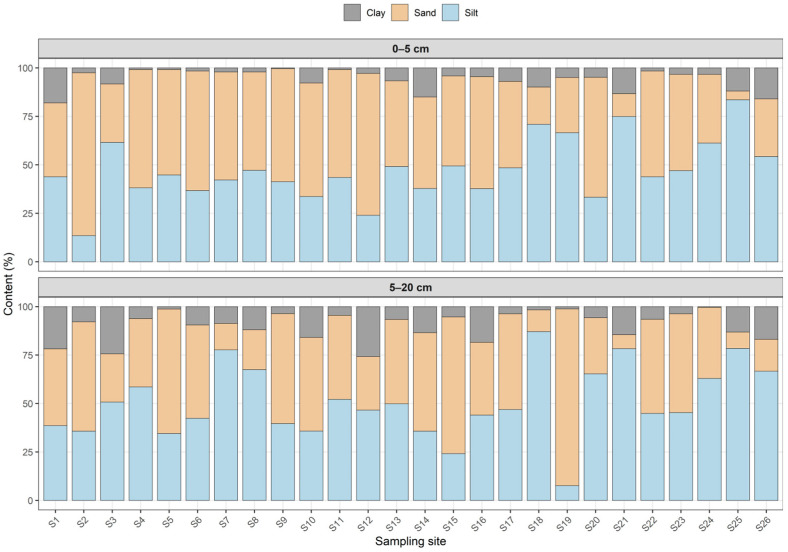
Ferrers diagram of the ranked distribution of the fine soil fraction (<0.01 mm) across the sampling sites in the Ridder industrial area (East Kazakhstan Region). The diagram compares the fine fraction content in two soil horizons (0–5 cm and 5–20 cm), illustrating spatial variability and vertical differentiation of soil texture within the investigated soil profiles.

The particle-size composition of soils within the study area exhibited pronounced spatial variability in both investigated horizons (0–5 and 5–20 cm). Overall, the soils were dominated by sand and silt fractions, while clay generally constituted a smaller proportion of the total particle-size distribution (Figure 2).

In the surface horizon (0–5 cm), sand content varied substantially across the sampling sites, ranging from only 4.49% at site S25 to more than 80% at site S2, indicating the coexistence of extremely coarse and relatively fine-textured soils within the investigated territory. Most soils were characterized by moderate proportions of sand and silt, forming sandy loam and loam textures. In contrast, several sites with elevated silt contents, such as S21 and S25, demonstrated distinctly finer soil structure, where the silt fraction exceeded 70%.

The clay fraction in the surface layer generally remained relatively low, typically below 10%. However, locally increased clay contents were observed at sites such as S1 and S26, where clay reached approximately 16–18%, indicating the presence of heavier loamy soils compared with the predominantly sandy substrates observed at sites such as S2 and S4 able S4.

In the subsurface horizon (5–20 cm), the variability of soil texture remained high. Sand content ranged from less than 10% at S21 to more than 90% at S19, highlighting the strong heterogeneity of soil materials within the study area. At the same time, silt remained a dominant fraction in many soils, particularly at sites characterized by loamy textures, whereas clay content locally exceeded 20–25% in several profiles, indicating a tendency toward heavier soil textures with depth.

The proportion of fine particles (<0.01 mm), which plays a critical role in sorption processes and heavy metal retention, also demonstrated considerable variability. In the surface layer, the content of this fraction ranged from only 6.55% at S2 to more than 54% at S26. A similar pattern was observed in the deeper horizon, where the fine fraction varied from 3.22% at S19 to nearly 60% at S25.

These contrasts indicate that soils with very low fine-particle content are predominantly sandy, whereas soils with elevated fine fractions correspond to heavy loam and clayey textures.

Overall, the obtained results demonstrate that the soils of the investigated area are characterized by substantial heterogeneity in particle-size distribution and texture class. The coexistence of sandy, loamy, and clay-rich soils within a relatively limited spatial area reflects the combined influence of natural soil-forming processes and long-term technogenic disturbances associated with intensive mining and metallurgical activity.

#### 2.1.3. Spatial Distribution and Concentration of Heavy Metals in Soils

**Figure 3:**
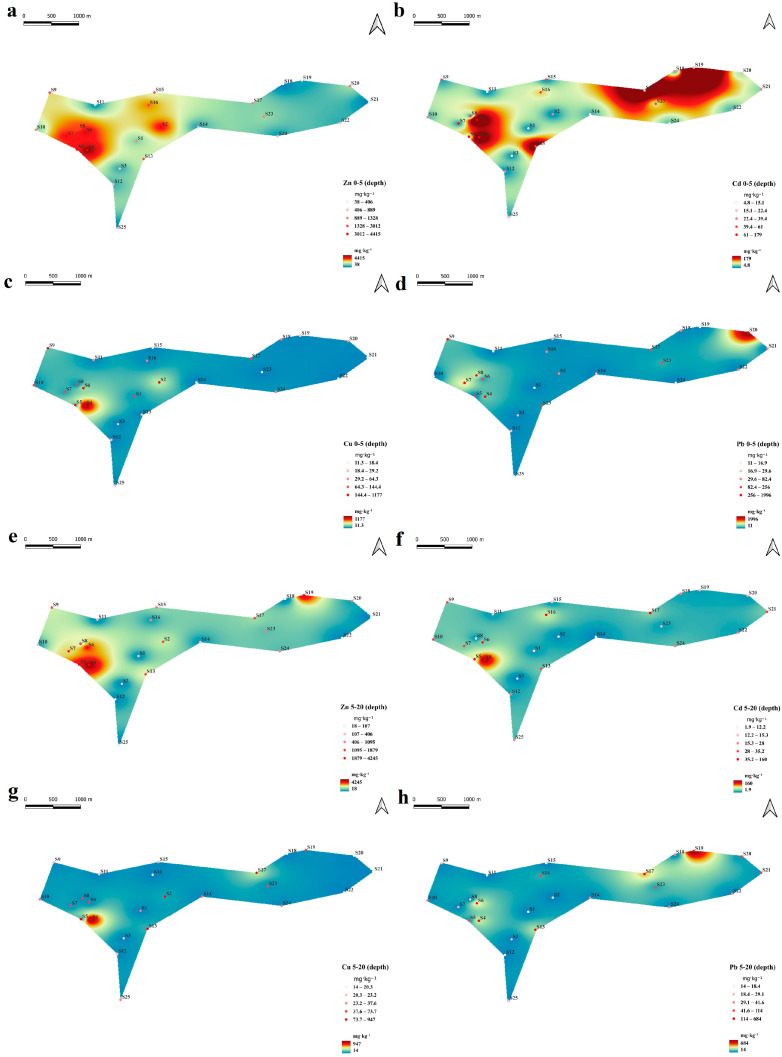
Spatial distribution of Zn, Cd, Cu, and Pb concentrations in soils of the study area interpolated. Panels (**a**–**d**) represent the surface soil horizon (0–5 cm), while panels (**e**–**h**) correspond to the subsurface horizon (5–20 cm). Sampling locations are indicated by red points.

The concentrations of Zn, Cu, Cd, and Pb in soils adjacent to the Ridder metallurgical plant exhibited pronounced spatial heterogeneity and clear enrichment within the industrial zone. Spatial interpolation maps indicate a distinct contamination hotspot located in the central part of the study area, whereas metal concentrations gradually decrease toward more distant locations (Figure 3).

Among the investigated metals, Zn was the dominant contaminant, reaching extremely high concentrations of up to 4415 mg·kg^−1^ in the surface soil and 4245 mg·kg^−1^ at 5–20 cm depth. Copper exhibited lower but still substantial concentrations, with maxima of 1177 mg·kg^−1^ in the surface horizon and 947 mg·kg^−1^ in the subsurface layer. Cadmium showed considerable enrichment at several sites, with maximum concentrations of 179 mg·kg^−1^ in the topsoil and 160 mg·kg^−1^ in deeper soils. Lead also demonstrated strong variability, reaching 1996 mg·kg^−1^ in the surface layer, while subsurface concentrations were generally lower but still elevated, with maxima up to 684 mg·kg^−1^ (Table S4).

**Figure 4:**
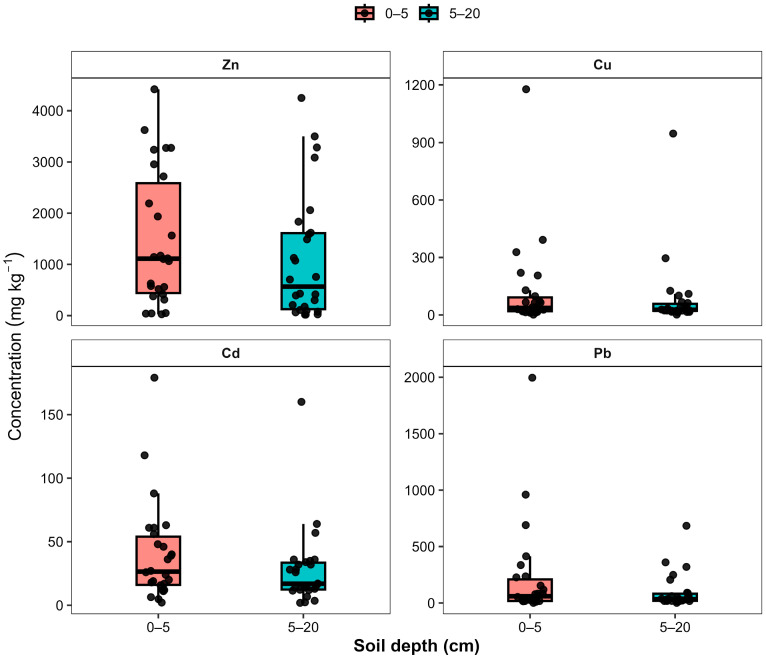
Boxplot distribution of heavy metals in soils at two depths (0–5 and 5–20 cm). Boxes represent the interquartile range, the central line indicates the median, whiskers show the data range, and points correspond to individual samples.

Boxplot analysis further highlights the substantial variability of metal concentrations across sampling sites (Figure 4). Zinc displayed the widest dispersion, with several extreme values exceeding 4000 mg·kg^−1^, indicating localized zones of intense contamination. Copper and Pb also showed pronounced outliers, particularly in the surface horizon, reflecting heterogeneous accumulation patterns across the study area. In contrast, Cd exhibited comparatively narrower interquartile ranges but still contained several elevated values corresponding to contamination hotspots.

Comparison between soil depths revealed that median concentrations of most metals were slightly higher in the surface horizon (0–5 cm), supporting atmospheric deposition as the primary contamination pathway. However, the presence of elevated values in the 5–20 cm layer indicates partial downward migration and long-term accumulation of heavy metals within the upper soil profile.

These results indicate that the investigated metals followed a consistent order of relative abundance: Zn > Pb > Cu > Cd, with Zn showing the strongest accumulation and the greatest spatial variability across the study area.

#### 2.1.4. Ecological Risk (*Eᵣ*) Assessment and Geoaccumulation Index (*I_geo_*)

**Figure 5:**
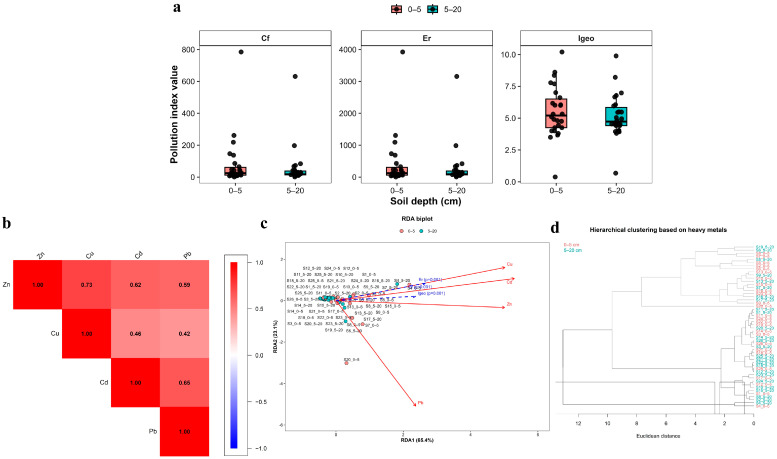
Statistical relationships and multivariate analysis of heavy metals in soils: (**a**) Distribution of pollution indices (*C_f_*, *Eᵣ*, and *I_geo_*) in soil samples; (**b**) Spearman correlation matrix among heavy metals (Zn, Cu, Cd, and Pb); (**c**) PCA biplot illustrating relationships between heavy metals and pollution indices; (**d**) Hierarchical clustering of soil samples based on heavy metal concentrations.

The calculated pollution indices demonstrated pronounced spatial variability across the investigated soil samples (Figure 5a). Both the contamination factor (*C_f_*) and ecological risk factor (*Eᵣ*) exhibited wide ranges of values, indicating heterogeneous levels of heavy metal accumulation within the study area. Several sampling sites were characterized by markedly elevated *C_f_* and *E_r_* values, reflecting localized zones of increased contamination intensity and ecological risk (*Eᵣ* ≥ 320). In contrast, the geoaccumulation index (*I_geo_*) showed comparatively lower variability, with most samples falling within moderate to strong pollution categories (1 < *I_geo_* ≤ 3). Differences between the two investigated soil depths (0–5 and 5–20 cm) were generally minor, suggesting relatively weak vertical differentiation of pollution indices and indicating that contamination processes influence both soil horizons in a similar manner (Table S5).

Spearman correlation analysis revealed predominantly positive relationships among the investigated heavy metals (Figure 5b), supporting the patterns observed in the distribution of pollution indices. The strongest correlation was detected between Zn and Cu (r = 0.73), followed by Cd and Pb (r = 0.65). Moderate positive correlations were also observed between Zn and Cd (r = 0.62) and Zn and Pb (r = 0.59), whereas weaker associations were found between Cu and Cd (r = 0.46) and Cu and Pb (r = 0.42). The predominance of positive correlations suggests that these metals share similar accumulation behavior and may originate from common anthropogenic or geochemical sources.

The multivariate analysis further confirmed these relationships. The ordination diagram indicated that Zn, Cu, and Cd were strongly aligned with the vectors of pollution indices (*C_f_*, *I_geo_*, and *Eᵣ*), highlighting their dominant contribution to the overall contamination level of soils (Figure 5c). The first two ordination axes explained the majority of the total variance, with RDA1 accounting for 65.4% and RDA2 for 23.1%, resulting in a cumulative explanation of 88.5% of the total variation. Sampling sites characterized by elevated pollution indices were positioned along the direction of the corresponding vectors, indicating areas with increased contamination intensity. These multivariate patterns are consistent with the positive correlations observed between metals in the Spearman analysis.

The hierarchical cluster analysis also supported the results obtained from correlation and ordination approaches (Figure 5d). The dendrogram grouped the sampling sites into several clusters based on similarities in heavy metal composition, reflecting spatial heterogeneity in contamination levels. Samples characterized by elevated metal concentrations tended to form distinct branches within the dendrogram, indicating sites with stronger anthropogenic influence (*Eᵣ* ≥ 160–320 and *I_geo_* ≥ 3–4). At the same time, the clustering pattern revealed groups of samples with similar geochemical characteristics across both soil depths, further supporting the observation that vertical differentiation of heavy metal contamination is relatively limited. The obtained results suggest that the consistency among correlation analysis, multivariate ordination, and cluster analysis indicates that the spatial distribution of soil contamination in the study area is largely controlled by a limited set of dominant heavy metals, particularly Zn, Cu, and Cd.

#### 2.1.5. Relationship Between Physiochemical Soil Properties and Heavy Metals

**Figure 6:**
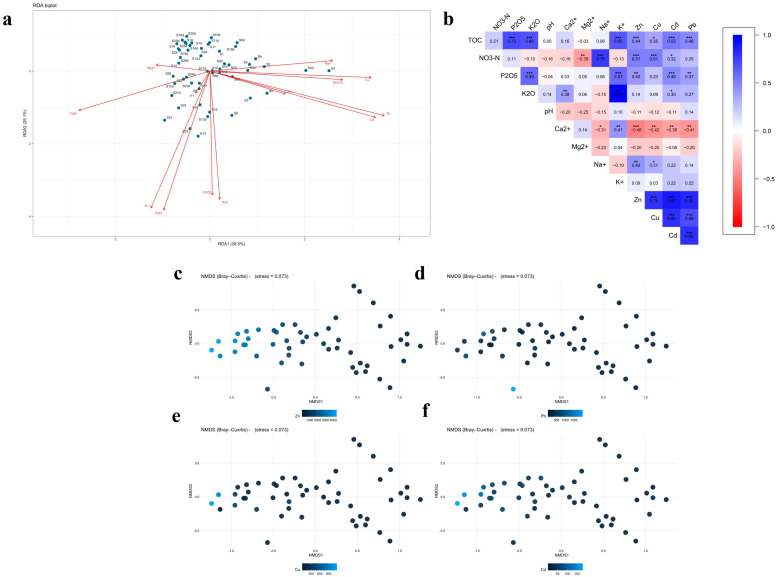
Relationships between physicochemical soil properties and heavy metal concentrations. (**a**) Redundancy analysis (RDA) illustrating the relationships between soil physicochemical properties and heavy metal concentrations, sampling sites are represented by blue points, and red vectors indicate environmental variables, with arrow length proportional to their influence and direction indicating correlation; (**b**) Spearman correlation matrix showing the relationships between soil chemical parameters and heavy metals; the color scale indicates the direction and strength of the correlations. Symbols (*, **, ***) denote levels of statistical significance (* *p* < 0.05; ** *p* < 0.01; *** *p* < 0.001); (**c**–**f**) Ordination plots of non-metric multidimensional scaling (NMDS) based on the Bray–Curtis index, demonstrating variations in community structure along concentration gradients of Zn (**c**), Pb (**d**), Cu (**e**), and Cd (**f**). The suffix “d” indicates samples collected from the deeper soil layer (5–20 cm).

PERMANOVA (ADONIS) analysis revealed a significant effect of the studied factor on the distribution of heavy metals across sampling sites, with a strong overall separation among groups (F = 17.92, *p* = 0.001). Redundancy analysis (RDA) was applied to quantitatively assess the relationships between soil chemical properties and heavy metal concentrations (Figure 6a). The first and second RDA axes explained 53.6% of the total variance, accounting for 28.5% (RDA1) and 25.1% (RDA2), respectively. The RDA1 axis was predominantly associated with Zn, Cd, Pb, and Cu, forming a distinct gradient of technogenic contamination. The vectors of NO_3_-N and Na^+^ were oriented in the same direction, further confirming their association with anthropogenic sources. In contrast, the vectors of Ca^2+^ and Mg^2+^ were directed oppositely, indicating their mitigating role in heavy metal accumulation. The RDA2 axis was strongly influenced by TOC content, P_2_O_5_, and K_2_O, reflecting a soil fertility gradient that is partially independent of metal contamination. The near-orthogonal orientation of fertility indicators relative to heavy metals suggests an incomplete overlap between the spatial patterns of soil fertility and contamination. The distribution of samples on the ordination diagram showed clustering in the central part, while several samples were shifted toward the heavy metal vectors, indicating the presence of localized contamination hotspots.

Spearman’s correlation analysis revealed clear and statistically significant relationships between soil chemical properties and the concentrations of heavy metals (Zn, Cu, Cd, and Pb), indicating a close coupling between soil fertility processes and metal accumulation (Figure 6b). TOC content showed strong positive correlations with P_2_O_5_ (r = 0.70; *p* < 0.001) and K_2_O (r = 0.66; *p* < 0.001), confirming the key role of organic matter in the retention and stabilization of nutrients within the soil matrix. In addition, TOC exhibited statistically significant positive correlations with Zn (r = 0.44; *p* < 0.001), Cu (r = 0.35; *p* < 0.05), Cd (r = 0.52; *p* < 0.001), and Pb (r = 0.46; *p* < 0.001), indicating the function of organic matter as an effective sorption pool for heavy metals under industrial impact conditions. Nitrate nitrogen (NO_3_-N) was positively associated with Na^+^ (r = 0.76; *p* < 0.001) and showed moderate correlations with Zn and Cu (r = 0.51; *p* < 0.001), suggesting a likely common anthropogenic origin related to industrial emissions and secondary atmospheric deposition. Soil pH was characterized by weak to moderate negative correlations with heavy metals, indicating increased metal mobility under more acidic conditions. The role of Ca^2+^ was particularly pronounced, as it exhibited significant negative correlations with Zn (r = −0.48; *p* < 0.001), Cu (r = −0.42; *p* < 0.01), Cd (r = −0.36; *p* < 0.01), and Pb (r = −0.41; *p* < 0.01). This highlights the importance of calcium in reducing metal bioavailability through competitive sorption and stabilization of soil structure. Mg^2+^ showed a similar but less pronounced trend. Strong positive correlations among Zn, Cu, Cd, and Pb (r = 0.69–0.87; *p* < 0.001) indicate a common source of contamination and similar geochemical behavior of these elements in the studied soils.

Taken together, these multivariate relationships indicate that the spatial distribution of heavy metals in the studied soils is controlled not only by their common technogenic origin but also by key soil physicochemical properties regulating their mobility and retention. In particular, the positive association with organic matter suggests that TOC acts as an important sorption pool for metals in contaminated soils, whereas the negative relationships with Ca^2+^ and Mg^2+^ indicate a buffering effect that may reduce metal mobility and bioavailability.

Non-metric multidimensional scaling (NMDS) based on the Bray–Curtis index demonstrated a stable ordination with a low stress value (0.073), confirming the reliability of the two-dimensional representation of the data (Figure 6c–f). The ordination was constructed using species composition data of the studied plant communities, whereas the concentrations of Zn, Pb, Cu, and Cd were fitted as environmental variables to illustrate their relationships with community structure. The NMDS ordination revealed a clear separation of samples along the NMDS1 axis in relation to increasing concentrations of Zn, Cd, Cu, and Pb. Samples with higher contamination levels were systematically shifted toward one region of the ordination space, indicating the key role of heavy metals in shaping differences among communities.

The most pronounced influence was exerted by Zn and Cd, which is consistent with their high mobility and phytotoxicity. The observed patterns reflect a shift in species composition toward metal-tolerant taxa under conditions of elevated contamination. The integrated analysis consistently demonstrates that heavy metal contamination acts as a dominant ecological filter determining soil chemical properties and the structure of plant communities. Organic matter enhances metal retention in soils, whereas Ca^2+^ and Mg^2+^ reduce their mobility and potential bioavailability. The close relationship between soil chemistry and community ordination confirms that plant communities in contaminated sites are primarily structured by edaphic constraints driven by metal accumulation, promoting the dominance of stress-tolerant and perennial species.

### 2.2. Plants

#### 2.2.1. Species Composition and Ecological Structure of Vegetation

The vegetation cover of the studied area is represented by a diverse assemblage of tree, shrub, and herbaceous species belonging to more than fifteen botanical families, among which *Salicaceae*, *Betulaceae*, *Poaceae*, *Asteraceae*, *Rosaceae*, and *Rubiaceae* are dominant (Table S6). Such taxonomic diversity reflects the mosaic nature of habitat conditions and the combined influence of natural factors and anthropogenic transformation associated with industrial activity.

The tree-shrub layer is formed mainly by perennial woody and shrub species. Representatives of the families *Salicaceae* (*Populus laurifolia*, *Populus balsamifera*, *Populus nigra*, *Salix viminalis*) and *Betulaceae* (*Betula pendula*, *Betula pubescens*) are characterized by moderate to relatively high Important Value indices (0.067–0.120), indicating their significant role in phytocenosis structure. Most of these species are confined to the industrial site, which suggests high tolerance to disturbed soil conditions and prolonged technogenic impact. In contrast, *Lonicera tatarica* (*Caprifoliaceae*) was recorded exclusively at the control site, reflecting its preference for less transformed habitats.

The herbaceous layer is characterized by the dominance of perennial species, primarily from the families *Poaceae*, *Asteraceae*, *Fabaceae*, and *Brassicaceae*, which account for the highest cumulative ecological importance. Perennial grasses (*Calamagrostis epigeios*, *Dactylis glomerata*, *Phleum phleoides*, *Poa pratensis*) exhibit high Important Value indices (0.110–0.135), reflecting their competitiveness and capacity to form stable communities under anthropogenically disturbed conditions.

Among forbs, representatives of the family *Asteraceae* are of particular importance. *Artemisia vulgaris* is characterized by the highest Important Value (0.140) and occurs at both the control and industrial sites, indicating high ecological plasticity. Several asteraceous species (*Tussilago farfara*, *Arctium tomentosum*, *Taraxacum officinale*, *Tripleurospermum perforatum*) were recorded exclusively at the industrial site and are classified as ruderal taxa typical of disturbed habitats. In contrast, meadow and forest-edge species (*Echinops tricholepis*, *Serratula coronata*, *Galatella hauptii*) were recorded only at the control area and are characterized by lower ecological importance values.

Representatives of the family *Fabaceae* (*Vicia sepium*, *Vicia cracca*, *Trifolium pratense*) show relatively high Important Value indices (0.104–0.121) and occur predominantly at the industrial site or at both sites, indicating their role in stabilizing vegetation cover through their ability to fix atmospheric nitrogen under stressful environmental conditions. The widespread occurrence of species such as *Urtica dioica* and *Convolvulus arvensis* further emphasizes the predominance of nitrophilous and ruderal perennials in the industrial area.

Overall, classification by life forms revealed a clear predominance of perennial herbaceous species, whereas annual and biennial plants were represented to a lesser extent. The distribution of Important Value indices and species presence across the control and industrial sites indicates that vegetation cover at the industrial site is formed mainly by ecologically plastic and stress-tolerant perennial species, whereas the control site is characterized by a higher contribution of meadow species and taxa less tolerant to anthropogenic impact.

#### 2.2.2. Heavy Metals Concentration in Dominant Agricultural Crops

The analysis of heavy metal concentrations in dominant agricultural crops revealed clear interspecific differences in both productivity and element accumulation patterns (Table 1).

Among the studied species, *Helianthus annuus* L. exhibited the highest aboveground biomass (44.25 g·plant^−1^), followed by *Triticum aestivum* L. (26.7 g·plant^−1^) and *Avena sativa* L. (20.55 g·plant^−1^), indicating species-specific growth responses under technogenic conditions.

**Figure 7:**
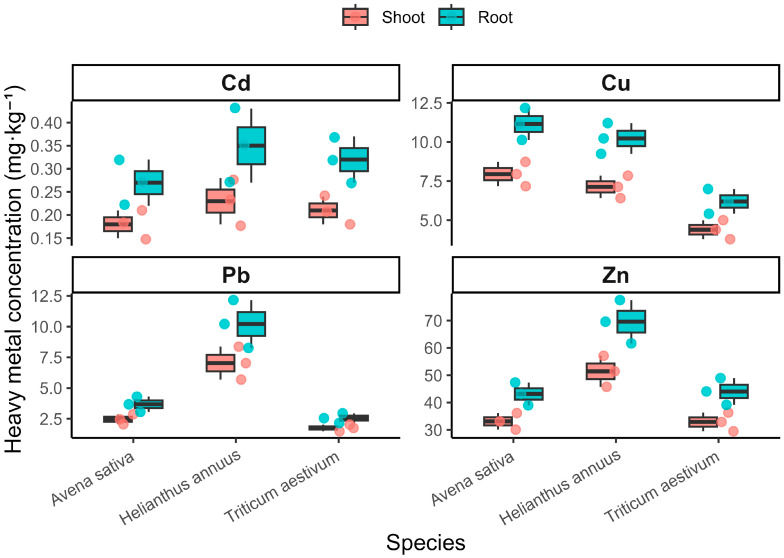
Box plots showing the distribution of heavy metal concentrations (Pb, Cd, Zn, and Cu) in shoots and roots of the studied crop species (*Avena sativa*, *Triticum aestivum*, and *Helianthus annuus*). Boxes represent the interquartile range (IQR), horizontal lines indicate medians, and whiskers denote the minimum and maximum values.

For all species, heavy metal concentrations were consistently higher in roots than in shoots, indicating preferential accumulation in the belowground organs. Across all experimental variants, metal concentrations followed a consistent decreasing order of Zn > Pb > Cu > Cd in both aboveground and belowground plant organs. The distribution patterns of heavy metal concentrations in shoots and roots of the studied crops are illustrated in the box plots (Figure 7).

As shown in Figure 7, the box plots clearly demonstrate a systematic predominance of heavy metal accumulation in root tissues across all studied plant species. Root concentrations of Zn and Pb are consistently higher than those observed in shoots, indicating limited upward translocation of metals within the plant. Moreover, the graphical representation highlights interspecific variability in metal uptake, with *Helianthus annuus* L. showing a tendency toward higher accumulation of Zn and Pb compared with the other crops. The relatively narrow interquartile ranges observed for some elements suggest stable accumulation patterns among replicates, whereas wider distributions indicate species-specific variability in metal uptake and internal transport.

In shoots, Zn concentrations ranged from 32.92 ± 3.41 mg·kg^−1^ (*Triticum aestivum* L.) to 51.45 ± 5.67 mg·kg^−1^ (*Helianthus annuus* L.), whereas root Zn concentrations were substantially higher, reaching 69.6 ± 7.92 mg·kg^−1^ in *Helianthus annuus* L. Similarly, Pb concentrations in roots exceeded those in shoots across all species, with maximum values observed in *Helianthus annuus* L. (10.21 ± 1.95 mg·kg^−1^). Cadmium concentrations remained comparatively low but followed the same pattern of greater root accumulation.

Copper also demonstrated pronounced root retention. In *Avena sativa* L., Cu concentrations reached 11.16 ± 1.02 mg·kg^−1^ in roots compared to 7.95 ± 0.78 mg·kg^−1^ in shoots, while in *Triticum aestivum* L. root Cu reached 6.2 ± 0.79 mg·kg^−1^.

Among the studied crops, *Helianthus annuus* L. showed the highest overall accumulation capacity for Zn and Pb, particularly in the root system. *Avena sativa* L. demonstrated relatively high Zn and Cu accumulation, whereas *Triticum aestivum* L. exhibited comparatively lower concentrations for most elements.

The predominance of metal retention in roots across all species indicates restricted translocation of metals to aerial parts and highlights the role of root tissues as the principal compartment of metal accumulation in the studied crops. At the same time, the present analysis is based on total metal concentrations in soils and plant tissues; therefore, the observed patterns primarily reflect general soil–plant transfer relationships rather than specific stabilization mechanisms occurring in the rhizosphere.

From the perspective of food security, the limited transfer of heavy metals to aboveground biomass is of particular importance. Reduced translocation to shoots and edible plant parts lowers the potential entry of toxic elements into the food chain, thereby mitigating risks to human health in technogenically impacted agroecosystems. Nevertheless, even moderate concentrations in edible tissues require continuous monitoring, as long-term cultivation on contaminated soils may pose cumulative risks.

#### 2.2.3. Bioconcentration and Translocation Factors in Dominant Agricultural Crops

**Figure 8:**
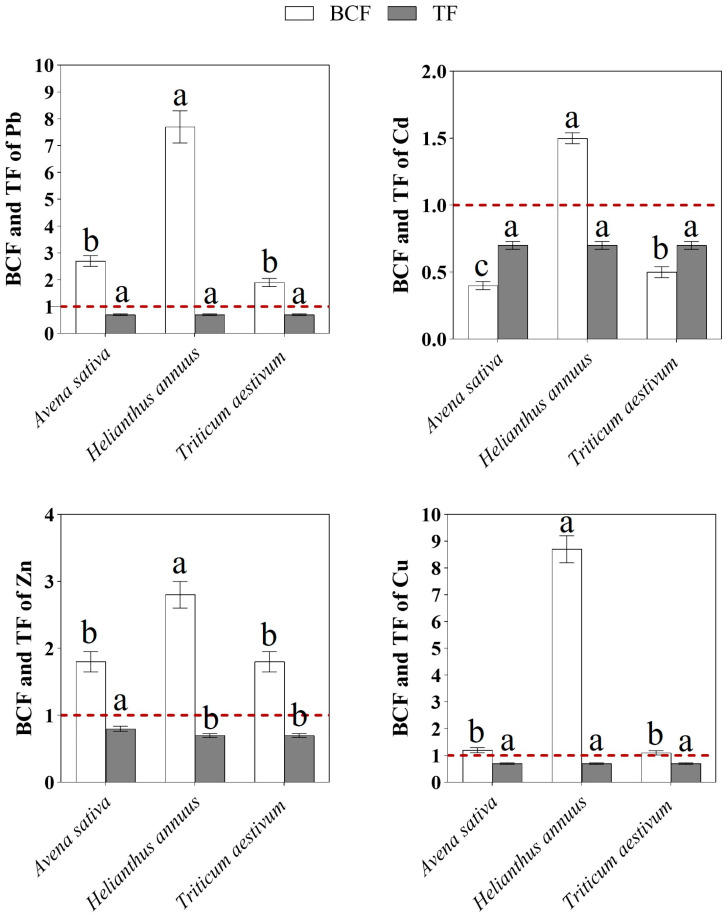
Bioconcentration factor (BCF) and translocation factor (TF) of four heavy metals found in agriculture crops. The data were showed for the means ± standard deviation with one-way analysis of variance. Different letters present significant statistical differences among different groups at the *p* < 0.05 level of Tukey’s HSD test, while identical letters indicate no significant difference. BCF-bioconcentration factor; TF-translocation factor. The red lines showed the threshold line of BCF and TF.

The results of the analysis of bioconcentration (BCF) and translocation factors (TF) revealed pronounced species-specific differences in the mechanisms of accumulation and redistribution of heavy metals (Pb, Cd, Zn, and Cu) in the studied agricultural crops (Figure 8). In all cases, TF values remained below unity, indicating limited translocation of metals from the root system to aboveground organs and confirming the dominant role of roots in metal retention.

For *Avena sativa* L., BCF values exceeded unity for Pb (2.7), Zn (1.8), and especially Cu (1.2), indicating a high capacity for accumulation of these elements in the root system. At the same time, the low BCF value for Cd (0.4) reflects limited root accumulation of cadmium; however, TF values for Cd (<1) indicate its effective immobilization in roots and weak transfer to shoots.

In *Helianthus annuus* L., the highest BCF values were recorded for Pb (7.7), Zn (2.8), and Cu (8.7), indicating a strong accumulation capacity of the root system in this species. Simultaneously, TF values for all metals remained at approximately 0.7, suggesting restricted transfer of metals to aboveground organs.

For *Triticum aestivum* L., BCF values for Pb (1.9) and Zn (1.8) exceeded unity, whereas moderate to lower BCF values were observed for Cd and Cu (0.5 and 15.1, respectively). Despite a comparatively lower accumulation capacity for Pb than *Helianthus annuus* L., *Triticum aestivum* L. exhibited a similar pattern of metal distribution, as evidenced by stable TF values (~0.7) for all elements.

Overall, interspecific comparison shows that root accumulation dominates over aboveground translocation for all studied metals, and the relative contribution of elements to bioconcentration decreases in the order Cu > Pb > Zn > Cd. The obtained results indicate that the studied crops are not effective phytoextractors but possess substantial phytostabilization potential, particularly with respect to copper and lead, which is of important significance for ecological risk assessment and management of contaminated soils.

## 3. Discussion

### 3.1. Soil Heterogeneity and Factors Governing Heavy Metal Dynamics

Previous studies have shown that technogenically disturbed territories are commonly characterized by pronounced heterogeneity of agrochemical and physicochemical soil properties, because anthropogenic geochemical anomalies are superimposed on the natural variability of soil cover [22,23]. The wide ranges recorded in the present study for TOC, available nitrogen, P_2_O_5_, K_2_O, and pH confirm that the soils of the investigated area are highly heterogeneous and spatially mosaic, which is typical of long-term industrially transformed ecosystems.

Among the analyzed parameters, the clearest vertical differentiation was observed for TOC and available nitrogen, both of which were enriched in the 0–5 cm layer. This pattern indicates preferential accumulation of organic residues and associated nutrient pools in the upper horizon, where atmospheric deposition, litter incorporation, and biological activity are most pronounced. In contrast, phosphorus and potassium exhibited weaker depth-related trends but stronger site-to-site variability, suggesting that their distribution is controlled not only by pedogenic processes but also by local edaphic and technogenic factors.

The relationships identified between organic matter and nutrient indicators are consistent with the recognized stabilizing role of the soil organo-mineral matrix [24]. In particular, the positive correlations of TOC with P_2_O_5_ and K_2_O indicate that organic matter functions as a major reservoir for both nutrient retention and sorption processes. This is especially important under industrial impact, where organic matter may simultaneously buffer soil chemistry and promote the retention of potentially toxic elements.

The observed pH range, from moderately acidic to near neutral, is also ecologically important because pH is one of the principal controls of heavy metal mobility in contaminated soils [25,26,27]. Under more acidic conditions, the solubility and potential mobility of Zn and Cd generally increase, whereas near-neutral conditions favor stronger sorption and reduced mobility. In this context, the relatively stable pH values observed between the two investigated depths suggest that vertical differences in metal redistribution are more likely controlled by organic matter, particle-size composition, and exchangeable cations than by strong pH stratification alone.

The exchange complex of the studied soils was dominated by Ca^2+^ and Mg^2+^, whereas Na^+^ and K^+^ represented only a minor fraction. Such a composition is typical of relatively buffered soil systems, in which alkaline earth cations occupy sorption sites and reduce the availability of trace metals for plant uptake [28]. The predominance of Ca^2+^ and Mg^2+^ in the present study therefore supports the interpretation that the soil exchange complex exerts a moderating influence on heavy metal mobility.

Finally, the generally low CaCO_3_ contents indicate that carbonate buffering plays only a limited role in the studied soils. Under such conditions, the retention of heavy metals depends predominantly on organo-mineral interactions rather than carbonate precipitation [29]. Consequently, the combination of elevated organic matter in the surface layer, weak carbonate buffering, and a Ca–Mg-dominated exchange complex appears to be a key factor controlling the retention, mobility, and vertical redistribution of metals in the investigated soil profiles.

### 3.2. Role of Soil Texture in Heavy Metal Retention and Migration

The studied soils exhibited substantial spatial variability in particle-size composition, ranging from sandy to clay-rich textures in both investigated layers. Such heterogeneity is typical of landscapes affected by both natural pedogenesis and long-term anthropogenic disturbance, where local redistribution of material, technogenic deposition, and profile mixing contribute to textural contrasts [30,31]. In the present study, this variability is particularly important because the proportion of fine particles strongly influences sorption capacity and, consequently, the behavior of heavy metals in soils.

Profiles enriched in silt and clay are expected to retain greater amounts of metals because fine fractions provide a larger specific surface area and more reactive sorption sites [30,31]. Clay minerals, in particular, contribute to the immobilization of Cd and Pb through adsorption and the formation of stable surface complexes [32,33]. The increase in fine material in several subsurface layers therefore offers a plausible explanation for the persistence of elevated metal concentrations below the surface, even where contamination is primarily driven by atmospheric deposition. Under such conditions, finer-textured horizons may act as secondary geochemical barriers that limit further downward migration and favor accumulation within the upper part of the profile [34,35].

By contrast, soils with a coarser texture and a low proportion of particles <0.01 mm are expected to have lower sorption capacity and weaker physicochemical buffering. This creates more favorable conditions for the downward movement of comparatively mobile metals, especially Cd and Zn [36,37]. The observed coexistence of coarse and fine-textured soils within the study area therefore provides an important explanation for the uneven vertical redistribution of contaminants and for the pronounced site-specific variability in heavy metal concentrations.

In general, the textural heterogeneity of the soils appears to be one of the main factors controlling the balance between retention and migration of heavy metals under technogenic impact. In this respect, the variability in fine fractions complements the effects of organic matter and exchangeable cations and helps explain why some profiles show stronger retention of contaminants in the upper horizon, whereas others exhibit more pronounced accumulation at depth [38].

### 3.3. Spatial Heterogeneity and Vertical Distribution of Heavy Metals

The concentrations of Zn, Cu, Cd, and Pb revealed extremely high spatial heterogeneity, with a distinct contamination hotspot in the central part of the study area and a gradual decrease toward more distant locations. This pattern is typical of soils affected by mining and metallurgical emissions, where atmospheric deposition, local accumulation of technogenic particles, and mechanical disturbance of soil cover generate strong small-scale variability [39,40]. The interpolation maps and boxplots indicate that contamination in the investigated area is not diffuse and uniform, but concentrated in specific sectors exposed to the strongest industrial influence.

Among the investigated metals, Zn was the dominant contaminant and showed the widest range of concentrations in both soil layers. Its extremely high concentrations in both the 0–5 and 5–20 cm horizons indicate not only intensive surface deposition but also prolonged accumulation within the upper soil profile. The persistence of high Zn values at depth suggests that vertical redistribution has occurred over time, likely promoted by profile disturbance, textural controls, and the movement of contaminated fine particles [41].

Copper displayed a similar spatial pattern but with lower absolute concentrations than Zn. Nevertheless, Cu also remained substantially enriched in several sites and, in some cases, showed marked increases with depth. Such behavior is consistent with the known affinity of Cu for organo-mineral components and its tendency to be retained in association with soil organic matter and fine particles [42,43]. This helps explain why high Cu contents were not confined strictly to the surface horizon.

Cadmium deserves particular attention because, despite lower absolute concentrations compared with Zn and Pb, it showed substantial enrichment and persistence in both horizons. This pattern is especially important from an ecological perspective, given the relatively high mobility and toxicity of Cd in contaminated soils [44,45]. The continuity of elevated Cd concentrations between the surface and subsurface layers indicates that this element is not restricted to recent deposition but participates in longer-term redistribution processes within the profile.

Lead exhibited the clearest contrast between horizons, with generally higher values in the surface layer, which is consistent with its relatively low mobility and strong association with solid soil phases. At the same time, isolated subsurface anomalies indicate that local burial of contaminated material or technogenic mixing has occurred at some sites [46]. Therefore, while atmospheric deposition appears to be the principal source of Pb enrichment, the present data also indicate that post-depositional redistribution has modified its vertical pattern at selected locations.

Taken together, the observed abundance sequence (Zn > Pb > Cu > Cd), the hotspot-like distribution, and the persistence of elevated concentrations in both soil layers indicate that the soil contamination pattern in the study area reflects long-term, spatially heterogeneous industrial impact superimposed on variable soil properties. The results further suggest that both source intensity and edaphic controls contribute to the present-day distribution of metals.

### 3.4. Ecological Risk Assessment and Metal-Specific Contributions

The calculated contamination factor (*C_f_*), geoaccumulation index (*I_geo_*), and ecological risk factor (*Eᵣ*) demonstrate that the heavy metal burden of the investigated soils is not only elevated in absolute terms but also ecologically significant. The marked spatial variability of these indices confirms that the study area contains both moderately contaminated sectors and localized hotspots characterized by very high ecological risk. In general, index values were higher in the 0–5 cm layer, which is consistent with the dominant role of atmospheric deposition in forming the present contamination pattern [47,48].

Cadmium emerged as the most critical contaminant when the data were evaluated through the risk-based framework of Hakanson. Although Zn often dominated in concentration, Cd generated the highest ecological risk because its toxicity coefficient strongly amplifies the significance of its enrichment [49]. The extremely high *Eᵣ* and *I_geo_* values recorded for Cd indicate that this element is the principal driver of ecological hazard in the investigated soils. Its persistence in the 5–20 cm layer further suggests that risk is not restricted to the immediate surface but extends into the upper profile, which is consistent with the recognized mobility and bioavailability of Cd under contaminated conditions [50,51].

Lead also contributed substantially to ecological risk, particularly in the most contaminated hotspots. The very high Pb-derived *Eᵣ* values recorded at selected sites indicate that localized lead enrichment can generate extreme ecological pressure even where its vertical mobility is generally limited. The strong spatial contrasts observed for Pb are consistent with the episodic and hotspot-driven character of industrial emissions in disturbed landscapes [52].

By contrast, Zn and Cu, despite their substantial concentrations, made a relatively smaller contribution to the overall risk pattern because of their lower toxicity weighting. Zinc generally fell within moderate to considerable risk classes, whereas Cu mostly remained within low to moderate categories, except at isolated sites. This difference highlights the importance of combining concentration-based indices with toxicity-sensitive ecological metrics when assessing contaminated soils.

Taken together, the ranking of ecological importance derived from the indices (Cd > Pb > Zn > Cu) is ecologically more informative than the ranking based solely on concentration. It indicates that the most hazardous components of technogenic contamination are not necessarily the most abundant ones, but those combining strong enrichment with high toxicity and appreciable mobility [52,53]. The persistence of very high-risk values in both horizons at several sites also suggests that remediation and management strategies should not focus exclusively on surface contamination but consider the upper profile as a whole.

### 3.5. Associations Between Soil Physicochemical Characteristics and Heavy Metal Distribution

The multivariate analyses showed that the distribution of heavy metals across the study area is structured rather than random and is jointly controlled by technogenic inputs and soil physicochemical properties. The significant PERMANOVA result and the high proportion of explained variance indicate that contamination patterns are organized along clear environmental gradients. Within this framework, Zn and Cd responded most strongly to the analyzed factors, whereas Cu and Pb showed somewhat lower but still significant sensitivity.

The RDA results indicate that the dominant gradient in the dataset is a technogenic contamination axis defined by Zn, Cd, Pb, and Cu, while a second, partially independent gradient reflects soil fertility variables. The orientation of NO_3_-N and Na^+^ in the same direction as the metal vectors suggests that these parameters are linked to anthropogenic disturbance and may co-vary with industrial deposition [54,55]. In contrast, the opposite orientation of Ca^2+^ and Mg^2+^ implies a buffering role, whereby these cations reduce metal mobility and weaken the expression of contamination in the soil solution.

The correlation structure refines this interpretation. Positive correlations between TOC and heavy metals indicate that organic matter acts as an important sorption pool under industrial impact, favoring the retention of Zn, Cu, Cd, and Pb in the solid phase. At the same time, the negative correlations between Ca^2+^ and all studied metals suggest that calcium-rich exchange complexes help stabilize the soil system and reduce the potential availability of contaminants. These results imply that organic matter in the investigated soils functions primarily as a sink rather than a source of heavy metals, at least at the scale resolved by the present dataset.

The multivariate relationships also provide insight into vertical redistribution. The enrichment of the upper horizon in TOC and nitrogen, together with the positive relationship between TOC and metal concentrations, suggests that the surface layer is a major retention zone for contaminants. However, the persistence of elevated metal concentrations in the subsurface horizon indicates that this retention is incomplete and that downward transfer occurs in part through the movement of fine particles, local profile disturbance, and element-specific mobility. In this context, Cd and Zn appear more responsive to redistribution processes than Pb.

The NMDS analysis extends these soil–chemical relationships to the plant community level. The clear separation of samples along the contamination gradient indicates that heavy metals act as a dominant ecological filter structuring vegetation in the technogenic landscape [56]. The especially strong influence of Zn and Cd is consistent with their known phytotoxicity and helps explain the shift toward stress-tolerant taxa in the more contaminated sites [57,58]. Thus, the ordination results are not merely visual summaries but show that metal contamination, moderated by organic matter and base cations, has direct ecological consequences for both soil functioning and vegetation structure.

### 3.6. Plant Community Structure in Technogenically Disturbed Vegetation

The floristic composition of the investigated area reflects the combined influence of habitat heterogeneity and long-term industrial transformation. The predominance of species belonging to *Salicaceae*, *Betulaceae*, *Poaceae*, *Asteraceae*, and *Fabaceae* indicates that the vegetation cover is structured by taxa capable of tolerating disturbed soils and fluctuating edaphic conditions. Similar taxonomic patterns have been reported for industrially impacted landscapes, where plant communities are dominated by ecologically plastic species capable of tolerating both mechanical disturbance and chemical stress associated with soil contamination [59]. The clear dominance of perennial herbs and the widespread occurrence of ruderal and nitrophilous species at the industrial site therefore reflect vegetation reassembly under conditions of chronic anthropogenic pressure.

Vegetation surveys were conducted at the same locations as the 26 soil sampling sites, allowing plant community composition to be directly related to the spatial distribution of soil properties and heavy metal contamination. The designation of the industrial site refers to areas situated within the zone of direct influence of the Ridder metallurgical complex, where long-term industrial emissions have resulted in elevated concentrations of heavy metals in soils. Under such conditions, vegetation structure is commonly shaped by the combined effects of physical disturbance and edaphic stress caused by metal accumulation [60].

The high Important Value indices recorded for perennial grasses and for Artemisia vulgaris indicate that ecological plasticity and tolerance to disturbance are key determinants of community dominance in the studied area. Species with broad ecological amplitude and efficient stress-tolerance mechanisms are often able to persist in contaminated environments, whereas more sensitive taxa decline or disappear along pollution gradients. This pattern has been widely documented in metalliferous ecosystems, where tolerant species progressively replace less adapted taxa under conditions of long-term environmental stress [61]. Consequently, taxa restricted to the control site are likely less tolerant to technogenic influence and therefore less competitive under contaminated conditions. This contrast between industrial and control sites supports the interpretation that industrial pressure acts as a strong ecological filter shaping species composition.

The vegetation data are also consistent with the NMDS results, which indicate that changes in community structure follow the gradient of heavy metal contamination identified in the soil analyses. Similar relationships between vegetation structure and soil contamination gradients have been reported in numerous studies of polluted industrial landscapes. The shift toward perennial, ruderal, and stress-tolerant species suggests that plant communities in the industrial zone are assembled not only by general disturbance processes but also by edaphic stress associated with metal accumulation. Consequently, the observed floristic pattern can be interpreted as a biological expression of the soil contamination gradients identified in the chemical analyses.

Taken together, the observed floristic patterns indicate that vegetation responses in the study area are closely linked to the spatial distribution of heavy metals in soils. The dominance of disturbance-tolerant and ruderal species reflects adaptive strategies that enable plant communities to persist under conditions of long-term technogenic stress. At the same time, the reduced representation of sensitive taxa indicates that industrial contamination may lead to gradual simplification of plant community structure and a decline in local biodiversity, a phenomenon frequently reported for polluted industrial ecosystems [61].

### 3.7. Levels of Heavy Metals in Dominant Agricultural Crops

The crop data demonstrate clear species-specific differences in both biomass production and metal accumulation. *Helianthus annuus* L. combined the highest aboveground biomass with the highest concentrations of Zn and Pb, particularly in roots, indicating relatively high tolerance to contaminated soil conditions and a greater capacity to accumulate metals in belowground tissues. By comparison, *Avena sativa* L. and *Triticum aestivum* L. showed lower biomass and generally lower concentrations of most elements, suggesting more conservative uptake behavior.

A common feature across all crops was the predominance of root over shoot accumulation. This pattern was evident for all studied metals and was especially pronounced for Pb and Zn, indicating restricted upward transport from belowground to aboveground organs. The BCF values further support species-specific differences in accumulation capacity, whereas the consistently low TF values indicate that internal translocation to shoots remained limited. However, TF < 1 should be interpreted primarily as evidence of restricted translocation rather than as direct proof of phytostabilization mechanisms. In the absence of data on bioavailable soil fractions, metal speciation, or rhizosphere processes, the present results are more appropriately interpreted as reflecting general soil–plant transfer relationships than demonstrating a specific stabilization mechanism [62].

From an agricultural perspective, the limited transfer of metals to aboveground biomass is important because it reduces the immediate risk of entry of contaminants into the food chain. At the same time, the measurable concentrations of Pb and Cd in shoots indicate that food safety concerns cannot be disregarded, especially under long-term cultivation in technogenically affected soils. Thus, the crop results highlight an important duality: the studied species restrict translocation to aerial parts, yet they still remain exposed to contaminated soil conditions and may contribute to chronic human exposure if agricultural use is maintained without monitoring.

In this context, comparison with internationally accepted food safety standards and regulatory limits becomes essential for evaluating the real-world implications of crop cultivation in contaminated soils [44]. Maximum permissible concentrations for toxic elements such as Cd and Pb in food products are strictly regulated in many countries and are typically set at very low levels due to their cumulative toxicity and potential health effects [52]. Even moderate concentrations of these metals in edible plant tissues may therefore represent a potential pathway of human exposure through the diet. Consequently, the presence of detectable Pb and Cd in aboveground plant organs suggests that agricultural production in technogenically affected landscapes should be accompanied by systematic monitoring of crop quality and careful assessment of compliance with food safety regulations [48]. Such an approach allows environmental contamination to be directly linked to potential human health risks and provides a more realistic framework for evaluating the sustainability of agricultural use in polluted regions.

The interspecific differences observed in BCF and TF also suggest that crop species differ in their practical suitability for contaminated agroecosystems. *Helianthus annuus* L. showed particularly strong root accumulation of Pb, Zn, and Cu, whereas *Avena sativa* L. also retained appreciable quantities of Zn and Cu in roots. *Triticum aestivum* L. displayed a similar general pattern of root retention but with lower overall accumulation. These differences indicate that the studied crops behave more as excluder-type plants with limited shoot transfer than as effective phytoextractors [63,64].

In general, the crop data do not support intensive phytoextraction as the dominant response. Rather, they indicate that the principal feature of the studied species is restricted translocation to shoots combined with substantial retention in roots. This pattern is ecologically relevant because it may lower short-term transfer to edible biomass, but it does not in itself demonstrate rhizosphere stabilization or reduced ecological risk. Therefore, the agricultural significance of these findings lies mainly in risk management: cultivation in contaminated soils may remain possible only under careful monitoring of edible tissues and with explicit consideration of long-term soil-to-plant transfer.

## 4. Practical Implications

The results obtained in this study demonstrate that long-term mining and metallurgical activity in the city of Ridder has led to the formation of persistent technogenic geochemical anomalies in soils, characterized by extremely high concentrations of Zn, Pb, Cu, and Cd and the formation of localized contamination hotspots. Such soils function as long-term reservoirs of potentially toxic elements and represent a potential ecological and sanitary risk for agricultural production, vegetation development, and land use in industrially transformed territories.

From a practical perspective, the spatial heterogeneity of contamination revealed in this study highlights the importance of site-specific environmental monitoring and risk assessment. The identification of contamination hotspots and areas with comparatively lower metal concentrations provides a basis for zoning contaminated territories and prioritizing remediation measures. In particular, areas characterized by extremely high ecological risk values (*Eᵣ* ≥ 320), primarily associated with Cd and Pb contamination, should be considered priority zones for environmental management and land-use regulation.

The results also demonstrate that soil physicochemical properties significantly influence the mobility and retention of heavy metals. The positive relationship between organic matter and heavy metal concentrations indicates that TOC acts as an important sorption pool, contributing to the immobilization of metals in the soil matrix. At the same time, the negative correlations with Ca^2+^ and Mg^2+^ highlight the stabilizing role of base cations in reducing metal mobility and bioavailability. These relationships suggest that soil management practices aimed at maintaining organic matter content and improving the base saturation of soils may contribute to reducing metal mobility in contaminated agroecosystems.

The analysis of agricultural crops further provides practical insights into soil–plant interactions under technogenic conditions. All studied crops demonstrated limited translocation of heavy metals to aboveground organs (TF < 1), indicating restricted movement of metals from roots to shoots. This pattern suggests that these crops function primarily as metal excluders, retaining contaminants predominantly in root tissues. From an agricultural perspective, this characteristic may reduce the immediate transfer of heavy metals into edible plant parts, although continuous monitoring of crop products remains essential to ensure food safety.

Finally, the identification of locally adapted plant species capable of tolerating elevated metal concentrations provides an important basis for developing regionally appropriate phytoremediation strategies. Native perennial species dominating the vegetation cover of the industrial zone may contribute to the stabilization of contaminated soils and reduction in metal mobility within the soil–plant system. Therefore, the integration of phytostabilization approaches with soil management practices and environmental monitoring programs represents a promising direction for the ecological rehabilitation and sustainable land use of technogenically disturbed territories in the Ridder region.

## 5. Materials and Methods

### 5.1. Study Area

The study was conducted in the adjacent area of the city of Ridder, East Kazakhstan region (50°20′21″ N, 83°30′21″ E), one of the largest mining and metallurgical centers of the Republic of Kazakhstan (Figure S1). The administrative territory of Ridder covers 3390 km^2^. The city forms part of KazZinc LLP as an independent subdivision of Kazakhstan’s non-ferrous metallurgical industry. Since 1 February 1997, KazZinc has operated as an integrated production and economic complex.

**Figure 9:**
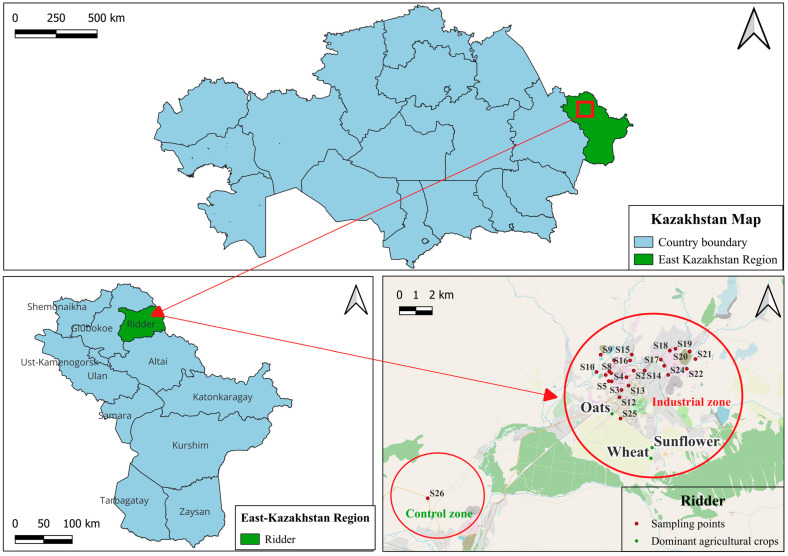
Distribution of the sample locations in the study area.

Ridder has a long history of intensive industrial development associated with the extraction and processing of polymetallic ores, primarily zinc, lead, and precious metals. The city-forming enterprise is KazZinc LLP-Ridder Mining and Processing Complex, which includes three mines, lead and zinc smelters, a concentrator plant, and a mechanical repair facility. These facilities represent the principal anthropogenic sources of heavy-metal inputs to the environment. Ridder is located in the mountainous part of the Altai region and is characterized by complex topography, which markedly influences atmospheric transport and the deposition of pollutants (Figure 9).

The local climate is sharply continental, with cold winters, moderately warm summers, and relatively high precipitation. Mean annual precipitation within the city is 300–600 mm, whereas in the surrounding mountainous areas it can reach up to 900 mm year^−1^, thereby promoting the active migration of heavy metals, enhancing surface runoff, and contributing to secondary soil contamination.

### 5.2. Soil Sampling and Analyses

Soil sampling was carried out using the envelope (diagonal composite) method, in which five subsamples were collected diagonally across each sampling plot and combined into a composite sample. A total of 26 sampling sites were investigated, and soil samples were collected from two depths: 0–5 cm and 5–20 cm. These two soil depths were selected to differentiate between the uppermost soil layer strongly influenced by atmospheric deposition of technogenic pollutants (0–5 cm) and the underlying root-inhabited horizon where plant–soil interactions and element migration processes occur (5–20 cm). The geographic coordinates of the sampling sites were recorded using a global positioning system (GPS) device. Detailed geographic and environmental characteristics of the sampling sites, including coordinates, mesotopography, parent material, soil type, vegetation, and functional zone, are provided in Table S7. Soil types were identified according to the World Reference Base for Soil Resources (WRB). The mass of each individual subsample did not exceed 200 g, while the total mass of each composite sample was at least 1 kg, ensuring its representativeness for subsequent laboratory analyses. In total, 52 soil samples were collected and used for further physicochemical analyses.

TOC content was determined by the potassium dichromate oxidation method in sulfuric acid medium according to State Standard (GOST) 26213-91 [65]. The analysis was based on oxidation of organic carbon using potassium dichromate (K_2_Cr_2_O_7_) in concentrated sulfuric acid (H_2_SO_4_), with the excess dichromate determined by titration with ferrous sulfate solution using standard laboratory titration equipment (automatic burette system, Brand GmbH, Wertheim, Germany) [66]. The carbonate content was determined using the Kappen method [67]. Soil pH was measured potentiometrically using a laboratory pH meter (SevenCompact S220, Mettler Toledo, Greifensee, Switzerland) according to State Standard 26423-85 in a 1:2.5 soil-to-1 M KCl suspension [68]. Soil texture was determined using the Kachinsky pipette sedimentation method, a classical particle-size analysis technique based on the sedimentation principle according to Stokes’ law, allowing the quantification of sand, silt, and clay fractions [69]. Readily hydrolysable nitrogen was determined by the Tyurin–Kononova method. Mobile forms of phosphorus and potassium were determined using the Machigin method according to State Standard (GOST) 26205-91 [70].

The soil samples were collected and sieved. A quantity of 2 g of dried soil sample was transferred into a 100 mL Erlenmeyer flask and concentrated nitric acid was added. Then, the sample was boiled in a water bath at 80 °C for 3 h. Next, it was cooled at room temperature, 25 mL water was added, and the resulting extract was filtered on filter paper into a 25 mL volumetric flask. These clear filtrate solutions were analyzed using an atomic absorption spectrometer [71]. HM concentrations in polluted soils exceeding the MPC were determined according to the regulations. MPC values (mg·kg^−1^) for HMs were 32.0 for Pb, 23.0 for Zn, 2.0 for Cd and 3.0 for Cu [72].

### 5.3. Plant Sampling and Analyses

To assess the natural biodiversity of the study area, geobotanical surveys were conducted at sites located near industrial facilities as well as at control locations situated at a distance from major sources of industrial impact. Vegetation sampling was performed during the peak vegetation period (June–July) within 1 m × 1 m plots established at each sampling site. All vascular plant species within the plots were recorded and identified using regional floristic guides of Kazakhstan, and the nomenclature followed the Plants of the World Online database. Plant species were classified according to their life forms (annuals, perennial herbs, and shrubs). The Importance Value Index (IVI) was calculated as the sum of relative abundance, relative frequency, and relative coverage for each species. Projective cover (%) was estimated proportionally from the Importance Value (IV) index and expressed as relative percentage contribution of each species within the plant community.

In addition, the dominant agricultural crops cultivated in the vicinity of industrial zones were identified, including oat (*Avena sativa* L.), sunflower (*Helianthus annuus* L.), and wheat (*Triticum aestivum* L.). Samples of these dominant crops were collected from both industrial and control sites. Plant samples were washed with distilled water, air-dried, and oven-dried at 65 °C to constant weight, after which the dried material was ground into a homogeneous powder.

An aliquot (1–2 g) of homogenized plant material (shoots and roots of *Avena sativa* L., *Helianthus annuus* L., and *Triticum aestivum* L., collected at the maturity stage) was placed in a 50 mL quartz crucible and subjected to dry ashing in a muffle furnace. The temperature was gradually increased to 500–550 °C and maintained for several hours until a homogeneous ash was obtained. After cooling, the ash was dissolved in 1% nitric acid, and the resulting solution was transferred to a volumetric flask and diluted to a final volume of 25 mL with distilled water prior to chemical analysis.

### 5.4. Atomic Absorption Spectrometry

Calibration curves were constructed for lead (Pb), zinc (Zn), cadmium (Cd), and copper (Cu) using certified atomic absorption stock solutions (1.0 g/L; Agilent Technologies, Santa Clara, CA, USA), prepared by successive dilution. Working calibration standards in the concentration range of 1–10 mg·L^−1^ were obtained using ultrapure water produced by a Milli-Q purification system (Merck Millipore, Darmstadt, Germany). All reagents were of analytical grade, including nitric acid (65%) and hydrogen peroxide (30%).

The analytical procedure was validated using reference standard solutions, with acceptable results required to fall within ± 1% of the certified values. Method validation included the evaluation of linearity and calibration range. Linearity was assessed by analyzing reference standard solutions in five independent replicates for each element. The limits of detection (LOD) and limits of quantification (LOQ) for Pb, Zn, Cd, and Cu were determined based on measurements at concentrations of 1, 2, 5, and 10 mg·L^−1^.

The concentrations of Pb, Zn, Cd, and Cu were determined using a flame atomic absorption spectrometer (Agilent 240FS, Agilent Technologies, Santa Clara, CA, USA) equipped with single-element hollow cathode lamps and an air-acetylene flame. The analytical wavelengths were 283.3 nm for Pb, 232.0 nm for Zn, 228.8 nm for Cd and 324.8 nm for Cu. The gas flow rate was maintained at 50 dm^3^/h, with an aspiration rate of 5 cm^3^/min. Single-element hollow cathode lamps (Agilent Technologies, Santa Clara, CA, USA) specific to each metal were used as radiation sources (Table S8).

### 5.5. Validation of the Method

Quantitative determination of heavy metals (HMs) was performed using external calibration, with calibration curves constructed over the concentration range of 1.0–10.0 mg·L^−1^. Each calibration curve was generated using four concentration levels. The calibration parameters, including correlation coefficients (R^2^), regression equations, linear ranges, limits of detection (LOD), and limits of quantification (LOQ) for each HM, are summarized.

All calibration curves exhibited excellent linearity, with correlation coefficients exceeding 0.9995, indicating a strong analytical response across the tested concentration range. The LOD values ranged from 0.04 to 0.08 mg·L^−1^, while the LOQ values varied between 0.12 and 0.24 mg·L^−1^, demonstrating the high sensitivity and suitability of the method for trace-level determination of heavy metals.

Table S9 presents the mean recovery values and corresponding standard deviations obtained using certified reference standard solutions for each heavy metal. The measured concentrations showed good agreement with the certified values at the 95% confidence level, confirming the accuracy of the analytical method. The mean recovery values ranged from 99.1% to 100.3% across all analyzed metals, with low standard deviations, indicating satisfactory precision and reliability of the method.

### 5.6. Sample Digestion

Approximately 0.5 g of homogenized sample was placed in a digestion vessel and treated with 5 mL of concentrated nitric acid (65%, analytical grade). The mixture was pre-digested at room temperature for 30 min and subsequently heated on a digestion block at 120 °C until near dryness. After cooling, 2 mL of hydrogen peroxide (30%) was added to complete oxidation of organic matter. The digest was diluted to a final volume of 25 mL with ultrapure water (Milli-Q system, Merck Millipore, Germany) and filtered prior to atomic absorption spectrometry analysis.

### 5.7. Ecological Risk Assessment and Geoaccumulation Index

The potential environmental risk index was proposed by Hakanson [8]. This method simultaneously takes into account several factors: concentration of HMs in soil, type of pollutant, and toxicity level. It comprehensively estimates the potential impact of HMs on environmental systems. It is possible to estimate the risk presented by a single factor (environmental risk coefficient, *E_r_*) as well as the risk presented by a number of elements (potential environmental risk index, *R_I_*). The risk is calculated using the following equations [73]:

*C_f_* = *C_sample_*/*C_background_* (1)

where *C_f_* is the contamination factor, *C_sample_* is the concentration of HMs in polluted soil, mg∙kg^−1^, and *C_background_* is the natural background concentration in the soil, mg∙kg^−1^;

*E_r_* = *T_r_* × *C_f_* (2)

(3) $$R_{I}=\sum_{i=1}^{n}E_{r}$$

where *R_I_* is the comprehensive potential ecological risk index, *E_r_* is the individual potential ecological risk index of HM, *T_r_* is the toxicity coefficient of the HMs (Pb-5, Zn-1, Cd-30, Cu-5), and *C_f_* is the pollution coefficient of the HMs.

The value of *R_I_* indicates the type and quantity of the pollution. The classification criteria proposed by Hakanson [8] for the *R_I_* and *E_r_* values are shown in Table S10.

Geoaccumulation index.

The geoaccumulation index, *I_geo_*, estimates the degree of soil contamination by heavy metals, and is calculated on the basis of the concentrations of the metal present in the soil, the geochemical background value of this metal, and a coefficient of 1.5 to take possible deviations into account [74,75]:

*I_geo_* = *log*_2_(*C_n_*/1.5 × *B_n_*) (4)

where *I_geo_* is the geoaccumulation index of the HM, *C_n_* is the concentration of the HM in the soil, *B_n_* is the geochemical background value of the HM, and 1.5 is a deviation coefficient (Table S11). The level of pollution is divided into 7 classes, ranging from no pollution to extremely high pollution.

### 5.8. Bioconcentration Factors (BCF) and Translocation Factors (TF)

The bioconcentration factor was used to determine the phytoremediation properties of plants. According to this, if the index value of the factor is <1, the plant is tolerant of HMs, and if it >1, it is a hyperaccumulator. The bioconcentration factor denotes the ability of the parts of the plant to elementally accumulate pollutants from the environment [76]:

*BCF = C_plant_/C_soil_* (5)

where *BCF* is the bioconcentration factor, *C_plant_* is the concentration of heavy metals in plant tissues (mg∙kg^−1^), and *C_soil_* is the concentration of heavy metals in the soil (mg∙kg^−1^).

The translocation factor *(TF)* is the value of the metal concentration in the aerial part of the plant in relation to the concentration in the root of the plant. This value is used to estimate the ability of plants to transfer HMs to their aerial parts [76]:

*TF = C_aerial_/C_roots_* (6)

### 5.9. Statistical Analysis

Statistical analyses and graphical visualizations were performed in R (version 4.5.1), while spatial interpolation and mapping were conducted in QGIS (version 3.40). Prior to analysis, all quantitative variables were standardized to remove scale effects.

Multivariate relationships between soil chemical properties and heavy metal concentrations were examined using redundancy analysis (RDA) implemented in the vegan package. Non-metric multidimensional scaling (NMDS) based on Bray–Curtis dissimilarity matrices was applied to explore patterns of similarity among sampling sites, and ordination quality was evaluated using stress values. Differences in soil chemical composition associated with heavy metals (Zn, Cd, Cu, and Pb) were further assessed using permutational multivariate analysis of variance (PERMANOVA) with 999 permutations.

Relationships among variables were evaluated using Spearman rank correlation analysis implemented in the *Hmisc* package (version 5.1-2), with significance levels set at *p* < 0.05. Hierarchical cluster analysis based on Euclidean distance and Ward’s linkage method was applied to classify soil samples according to heavy metal concentrations using the *stats* package (version 4.5.1).

Data distributions were visualized using boxplots generated with the *ggplot2* package (version 3.5.1), while soil particle-size composition was illustrated using Ferrers diagrams constructed with the *ggtern* package (version 3.4.2). Spatial distribution of heavy metals was mapped using inverse distance weighting (IDW) interpolation in QGIS. Interpolation was restricted to the polygon representing the study area, while the control sampling site located outside this polygon was excluded to improve the readability of the resulting maps. Differences in heavy metal concentrations in plants were assessed using two-way ANOVA followed by Tukey’s HSD test (*p* < 0.05).

## 6. Conclusions

This study provides an integrated assessment of soil contamination and soil–plant interactions in technogenically transformed landscapes of the Ridder industrial region (East Kazakhstan). The results revealed pronounced spatial heterogeneity of heavy metal contamination, with extremely high concentrations of Zn, Pb, Cu, and Cd and the formation of distinct contamination hotspots near the industrial zone. Although Zn exhibited the highest absolute concentrations, cadmium represented the most critical contaminant from an ecological perspective due to its high toxicity and mobility. Ecological risk assessment confirmed that several sites exceeded the threshold of very high risk (*Eᵣ* ≥ 320), indicating persistent technogenic anomalies within the soil profile.

The structure of the vegetation cover reflects the effects of chronic technogenic stress and is formed predominantly by stress-tolerant, perennial, and ruderal species adapted to unfavorable soil conditions. The analysis of agricultural crops demonstrated that heavy metal accumulation occurs mainly in the root system with limited translocation to aboveground organs, indicating the predominance of phytostabilization mechanisms.

At the same time, the presence of heavy metals in cultivated plants highlights the importance of comparing the obtained concentrations with established food safety thresholds and regulatory limits, since in contaminated areas adjacent to industrial sources the transfer of toxic elements into the food chain may represent a potential pathway of human exposure.

In this context, the application of phytostabilization strategies requires appropriate post-harvest management of plant biomass. Since the roots of phytostabilizing plants may accumulate considerable amounts of heavy metals, their handling and disposal should be carefully controlled to prevent the potential re-release of contaminants into the environment. Of particular practical importance is the inventory of locally adapted plant species, which may serve as a basis for the subsequent selection of promising plants for the development of phytoremediation and phytostabilization technologies. Overall, the obtained findings provide a scientific foundation for ecological risk assessment, optimization of environmental monitoring, and the development of sustainable management and reclamation strategies for anthropogenically contaminated areas.

## Figures and Tables

**Table 1:** Heavy metal concentrations in shoots and roots of agricultural crops (*n* = 18; mg·kg^−1^; mean ± SD).

Species	Biomass (g·Plant^−1^)	Heavy Metals Concentrations (mg·kg^−1^)							
		Shoot (mg·kg^−1^, Mean ± SD)				Root (mg·kg^−1^, Mean ± SD)			
		Pb	Cd	Zn	Cu	Pb	Cd	Zn	Cu
*Avena sativa* L.	20.55	2.46 ± 0.41 c	0.18 ± 0.03 c	33.15 ± 3.02 a	7.95 ± 0.78 b	3.68 ± 0.62 c	0.27 ± 0.05 c	43.19 ± 4.18 a	11.16 ± 1.02 b
*Helianthus annuus* L.	44.25	7.03 ± 1.34 b	0.23 ± 0.05 b	51.45 ± 5.67 a	7.13 ± 0.72 b	10.21 ± 1.95 b	0.35 ± 0.08 b	69.6 ± 7.92 a	10.23 ± 0.98 b
*Triticum aestivum* L.	26.7	1.75 ± 0.27 b	0.21 ± 0.03 b	32.92 ± 3.41 a	4.39 ± 0.61 b	2.55 ± 0.39 b	0.32 ± 0.05 b	44.09 ± 4.88 a	6.2 ± 0.79 b

Note: Data are means ± standard deviations. Different letters present significant statistical differences among different groups at the *p* < 0.05 level of Tukey’s HSD test, while identical letters indicate no significant difference.

## Data Availability

The original contributions presented in this study are included in the article/Supplementary Material. Further inquiries can be directed to the corresponding author.

## Supplementary Materials

The following supporting information can be downloaded at: https://www.mdpi.com/article/10.3390/plants15060983/s1, Table S1: Physicochemical properties of soils at the sampling sites; Table S2: Summary of soil physical properties (particle-size fractions and dominant soil texture) across the sampling sites; Table S3: Particle-size distribution of soils; Table S4: Contents of heavy metals in soils of the territory adjacent to the Ridder Plant; Table S5: Assessment of soil contamination by heavy metals at depths of 0–5 and 5–20 cm using the contamination factor (*C_f_*), geoaccumulation index (*I_geo_*), and potential ecological risk factor (*Eᵣ*) for sampling sites S1–S26; Table S6: Ecological importance of plant species at the control site and the industrial area; Table S7: Geographic coordinates and environmental characteristics of the soil sampling sites; Table S8: LOD and LOQ values and linear parameters for the HM calibration standards; Table S9: Reference standard certified and measured values and the recovery; Table S10: Interpretation of the value of the potential environmental risk indicator; Table S11: Classification of Soil Contamination Levels Based on the Geoaccumulation Index (*I_geo_*); Figure S1: Study area in the Ridder industrial region (East Kazakhstan).

## Abbreviations

The following abbreviations are used in this manuscript:

HMs	Heavy metals
MPC	Maximum permissible concentrations
RDA	Redundancy analysis
NMDS	Non-metric multidimensional scaling
BCF	Bioconcentration Factor
TF	Translocation Factor
*C_f_*	Contamination factor
*I_geo_*	Geoaccumulation index
*Eᵣ*	Potential ecological risk
CaCO_3_	Calcium carbonate
K_2_O	Exchangeable Potassium Oxide
P_2_O_5_	Available Phosphorus
Mg^2+^	Exchangeable Magnesium
Na^+^	Exchangeable Sodium
Ca^2+^	Exchangeable Calcium
NO_3_-N	Nitrate Nitrogen
Zn	Zinc
Pb	Lead
Cd	Cadmium
Cu	Copper
SD	Standard Deviation
