# Supplementary material for: Assessment of Heavy Metal Accumulation in Soils and Dominant Agricultural Crops in an Industrial Environment of Ridder, East Kazakhstan Region
Source: Plants (Basel). 2026 Mar 23;15(6):983. doi: 10.3390/plants15060983 (PMC13030110; doi:10.3390/plants15060983)
Supplement: Supplementary file 1 [file plants-15-00983-s001.zip › Supplementary tables S1-S11.pdf]

Table S1. Physicochemical properties of soils at the sampling sites

Sampling site	Depth, cm	Total organic carbon, %	Available nitrogen (N), mg·kg⁻¹	Available P_2O_5 (mg·kg⁻¹)	Exchangeable K_2O (mg·kg⁻¹)	pH	$CaCO_3$, (%)	Exchangeable base cations (mmol(+)/kg)			
								Ca^{2+}	Mg^{2+}	Na^{+}	K^{+}
S1	0-5	3.26	64.4	52	210	4.65	2.29	249.6	24.0	1.0	0.6
	5-20	1.71	64.4	34	150	3.35	0.39	355.2	14.4	0.4	0.8
S2	0-5	5.42	67.2	112	210	5.77	trace	48.0	28.8	1.0	0.8
	5-20	4.05	75.6	68	320	6.65	trace	201.6	14.4	1.0	1.9
S3	0-5	4.94	70.0	98	680	6.21	trace	249.6	57.6	1.0	7.1
	5-20	2.17	53.2	18	280	6.74	trace	249.6	33.6	1.0	1.1
S4	0-5	3.45	70.0	46	280	6.23	trace	28.8	33.6	1.0	1.2
	5-20	2.91	64.4	24	400	6.04	trace	67.2	57.6	1.0	2.2
S5	0-5	2.33	53.2	52	390	6.86	trace	144.0	19.2	1.0	1.6
	5-20	2.93	75.6	86	390	6.29	trace	163.2	33.6	1.0	2.3
S6	0-5	5.38	47.6	214	420	5.93	trace	67.2	76.8	1.0	2.9
	5-20	1.98	42.0	110	510	5.96	trace	153.6	33.6	1.0	5.1
S7	0-5	3.46	58.8	142	600	6.25	trace	211.2	33.6	1.0	6.6
	5-20	3.29	50.4	56	450	5.76	trace	225.6	48.0	1.0	3.7
S8	0-5	7.95	50.4	206	620	6.12	trace	244.8	57.6	1.0	6.8
	5-20	3.18	53.2	76	550	6.14	trace	196.8	48.0	1.0	4.5
S9	0-5	3.56	67.2	100	240	4.84	trace	57.6	52.8	1.0	0.5
	5-20	1.40	70.0	50	200	4.72	trace	100.8	24.0	1.0	0.1
S10	0-5	4.21	64.4	46	550	5.83	trace	196.8	48.0	1.0	4.2
	5-20	1.03	25.2	8	200	6.25	trace	115.2	28.8	1.0	1.1
S11	0-5	2.30	19.6	66	190	6.52	trace	86.4	57.6	1.0	0.4
	5-20	2.08	19.6	70	110	6.67	trace	76.8	38.4	1.0	1.1
S12	0-5	4.62	75.6	126	370	6.36	trace	144.0	19.2	1.0	3.4
	5-20	2.31	39.2	44	420	7.01	trace	192.0	33.6	1.0	1.8
S13	0-5	8.97	58.8	166	950	6.61	trace	254.4	38.4	0.6	14.7
	5-20	8.76	64.4	132	780	6.58	trace	249.6	52.8	0.6	9.3
S14	0-5	1.94	1.0	32	500	7.2	2.79	148.8	43.2	0.1	4.9
	5-20	2.24	1.0	38	210	7.13	0.84	158.4	62.4	0.6	0.5
S15	0-5	7.07	1.0	202	630	5.57	0.39	216.0	52.8	0.6	6.1
	5-20	4.12	1.0	178	500	5.48	2.27	230.4	86.4	0.1	4.4
S16	0-5	7.78	1.0	214	670	6.08	0.98	134.4	62.4	0.6	7.6
	5-20	1.03	1.0	68	320	5.81	0.52	182.4	62.4	0.6	2.0
S17	0-5	4.48	1.0	64	670	6.78	1.88	134.4	24.0	0.6	6.7
	5-20	4.23	1.0	36	490	6.97	3.47	120.0	28.8	0.1	3.7
S18	0-5	1.13	1.0	20	240	6.54	0.75	220.8	76.8	0.1	0.5
	5-20	1.07	1.0	16	210	6.3	0.84	230.4	57.6	0.6	0.7
S19	0-5	1.73	1.0	22	230	6.53	0.75	196.8	67.2	0.6	0.7
	5-20	1.97	1.0	32	110	7.17	1.14	19.2	14.4	0.6	0.4
S20	0-5	1.84	1.0	122	330	6.33	1.20	62.4	33.6	0.6	1.7
	5-20	1.68	1.0	40	200	5.55	0.75	192.0	115.0	0.6	0.6
S21	0-5	3.35	1.0	182	1060	6.34	0.75	249.6	48.0	0.6	15.8

	5-20	2.51	1.0	176	820	6.76	0.84	244.8	48.0	0.6	8.2
S22	0-5	7.23	1.0	52	380	6.05	0.84	249.6	62.4	0.6	6.0
	5-20	0.97	1.0	14	200	5.85	0.75	139.2	101.0	0.6	0.9
S23	0-5	11.29	1.0	136	800	5.94	0.84	278.4	43.2	0.6	11.7
	5-20	4.04	1.0	94	570	6.29	0.91	172.8	38.4	0.6	7.9
S24	0-5	5.06	1.0	238	750	6.55	1.14	230.4	33.6	0.5	8.2
	5-20	4.45	1.0	144	410	6.81	0.91	182.4	38.4	0.1	2.9
S25	0-5	2.25	1.0	40	270	5.83	0.98	273.6	48.0	0.6	1.6
	5-20	2.22	1.0	38	270	5.51	0.91	268.8	67.2	0.6	1.5
S26	0-5	2.73	1.0	32	660	5.81	0.75	254.4	43.2	0.6	8.7
	5-20	2.40	1.0	18	430	5.94	0.75	254.4	28.8	0.6	3.7

Table S2. Summary of soil physical properties (particle-size fractions and dominant soil texture) across the sampling sites

Sampling Site	Depth, cm	ODW* % H_2O	Fraction Content (% of Absolute Dry Soil)			Dominant texture
			Particle Size (mm)			
			Sand	Silt	Clay	
S1	0-5	2.44	38.09	43.87	18.04	Silt-dominant
	5-20	2.60	39.63	38.60	21.77	Sand-dominant
S2	0-5	2.24	84.04	13.50	2.46	Sand-dominant
	5-20	2.68	56.43	35.76	7.81	Sand-dominant
S3	0-5	3.28	30.11	61.62	8.27	Silt-dominant
	5-20	3.12	24.86	50.78	24.36	Silt-dominant
S4	0-5	2.36	61.08	38.10	0.82	Sand-dominant
	5-20	3.00	35.26	58.56	6.19	Silt-dominant
S5	0-5	2.60	54.41	44.76	0.82	Sand-dominant
	5-20	2.80	64.20	34.57	1.24	Sand-dominant
S6	0-5	3.00	61.65	36.70	1.65	Sand-dominant
	5-20	2.84	48.13	42.40	9.47	Sand-dominant
S7	0-5	4.28	55.70	42.21	2.09	Sand-dominant
	5-20	3.16	13.67	77.65	8.67	Silt-dominant
S8	0-5	4.28	50.69	47.22	2.09	Sand-dominant
	5-20	2.76	20.61	67.46	11.93	Silt-dominant
S9	0-5	1.22	58.29	41.30	0.41	Sand-dominant
	5-20	1.18	56.69	39.67	3.64	Sand-dominant
S10	0-5	2.58	58.53	33.67	7.80	Sand-dominant
	5-20	1.66	48.34	35.79	15.86	Sand-dominant
S11	0-5	1.54	55.72	43.47	0.81	Sand-dominant
	5-20	1.12	43.37	52.18	4.45	Silt-dominant
S12	0-5	1.56	73.18	23.97	2.84	Sand-dominant
	5-20	2.14	27.65	46.60	25.75	Silt-dominant
S13	0-5	3.16	44.24	49.15	6.61	Silt-dominant
	5-20	2.88	43.58	49.84	6.59	Silt-dominant
S14	0-5	1.64	47.13	37.82	15.05	Sand-dominant
	5-20	1.62	50.80	35.78	13.42	Sand-dominant
S15	0-5	2.96	46.41	49.46	4.12	Silt-dominant
	5-20	2.46	70.47	24.20	5.33	Sand-dominant
S16	0-5	2.34	57.81	37.68	4.50	Sand-dominant
	5-20	1.94	37.59	44.05	18.36	Silt-dominant
S17	0-5	1.86	44.57	48.50	6.93	Silt-dominant
	5-20	1.98	49.40	46.93	3.67	Sand-dominant
S18	0-5	2.96	19.21	70.90	9.89	Silt-dominant
	5-20	3.12	11.23	87.12	1.65	Silt-dominant
S19	0-5	2.72	28.45	66.61	4.93	Silt-dominant
	5-20	0.58	91.15	7.64	1.21	Sand-dominant
S20	0-5	1.54	61.81	33.31	4.88	Sand-dominant
	5-20	2.64	28.92	65.33	5.75	Silt-dominant
S21	0-5	3.40	11.80	74.95	13.25	Silt-dominant
	5-20	3.48	7.17	78.33	14.51	Silt-dominant
S22	0-5	3.28	54.51	43.84	1.65	Sand-dominant

	5-20	2.00	48.57	44.90	6.53	Sand-dominant
S23	0-5	3.86	49.66	47.02	3.33	Sand-dominant
	5-20	2.10	50.97	45.35	3.68	Sand-dominant
S24	0-5	3.32	35.46	61.23	3.31	Silt-dominant
	5-20	2.78	36.64	62.95	0.41	Silt-dominant
S25	0-5	2.84	4.49	83.57	11.94	Silt-dominant
	5-20	17.92	8.38	78.46	13.16	Silt-dominant
S26	0-5	2.74	29.67	54.29	16.04	Silt-dominant
	5-20	2.84	16.43	66.70	16.88	Silt-dominant

Note: *Oven dry weight

Table S3. Particle-size distribution of soils

Sampling site	Fractions <0.01 mm (%)	Soil Type	Fractions <0.01 mm (%)	Soil Type
	0-5 cm		5-20 cm	
S1	52.1	heavy loam	43.1	heavy loam
S2	6.5	sandy	26.3	light loam
S3	38.8	medium loam	56.2	clayey
S4	13.5	sandy loam	34.2	medium loam
S5	22.2	light loam	35.4	medium loam
S6	8.6	sandy	34.9	medium loam
S7	42.2	heavy loam	42.1	heavy loam
S8	20.5	light loam	47.3	heavy loam
S9	16.6	sandy loam	21.0	sandy loam
S10	29.9	light loam	32.5	medium loam
S11	17.5	sandy loam	17.4	sandy loam
S12	14.6	sandy loam	67.0	clayey
S13	33.8	medium loam	39.9	medium loam
S14	37.0	medium loam	34.2	medium loam
S15	24.7	light loam	25.8	light loam
S16	14.7	sandy loam	42.0	heavy loam
S17	20.4	light loam	35.9	medium loam
S18	50.3	heavy loam	44.6	heavy loam
S19	34.9	medium loam	3.2	sandy
S20	7.3	sandy	48.5	heavy loam
S21	50.1	heavy loam	53.0	clayey
S22	19.4	sandy loam	30.2	medium loam
S23	12.1	sandy loam	26.9	light loam
S24	28.9	light loam	22.2	light loam
S25	51.8	clayey	59.9	clayey
S26	54.3	clayey	57.6	clayey

Table S4. Contents of heavy metals in soils of the territory adjacent to the Ridder Plant (n = 156; mg·kg^{-1})

Sampling site	Depth, cm	Zn, mg·kg^{-1}	Cu, mg·kg^{-1}	Cd, mg·kg^{-1}	Pb, mg·kg^{-1}
S1	0-5	1106 ± 2.5	98 ± 1.5	11.1 ± 1.1	13.7 ± 2.3
	5-20	207 ± 2.6	23.3 ± 1.7	11.5 ± 1.2	17.6 ± 2.5
S2	0-5	3276 ± 1.3	392 ± 2.5	39 ± 1.3	82 ± 1.7
	5-20	1489 ± 2.2	101 ± 2.1	6.7 ± 1.0	14 ± 1.3
S3	0-5	578 ± 1.0	21.9 ± 1.2	6.4 ± 1.1	20.2 ± 2.1
	5-20	110 ± 3.0	21.3 ± 1.0	1.9 ± 0.5	18.5 ± 1.8
S4	0-5	4415 ± 1.5	1177 ± 2.4	179 ± 2.1	414 ± 2.1
	5-20	4245 ± 3.2	947 ± 2.0	160 ± 1.9	249 ± 2.4
S5	0-5	3275 ± 2.1	206 ± 1.8	63 ± 1.5	83 ± 2.0
	5-20	3284 ± 2.4	110 ± 2.1	64 ± 2.1	66 ± 1.8
S6	0-5	3624 ± 3.1	328 ± 3.5	118 ± 2.4	227 ± 2.6
	5-20	3086 ± 2.5	44.1 ± 1.6	36 ± 1.4	320 ± 2.7
S7	0-5	3239 ± 3.0	129 ± 1.4	88 ± 2.1	960 ± 2.6
	5-20	2060 ± 2.0	66.9 ± 1.0	35 ± 1.5	21.9 ± 2.4
S8	0-5	2955 ± 2.7	66.4 ± 1.0	61 ± 2.1	690 ± 2.6
	5-20	1588 ± 2.3	44.7 ± 1.1	12 ± 1.8	25 ± 1.2
S9	0-5	2191 ± 3.1	220 ± 1.6	61 ± 2.0	336 ± 2.5
	5-20	1618 ± 2.8	22.8 ± 1.0	32 ± 2.2	22.8 ± 1.3
S10	0-5	1117 ± 2.4	72 ± 1.4	36.1 ± 1.5	16.9 ± 1.0
	5-20	393 ± 3.1	33.2 ± 1.2	28.1 ± 2.1	35.8 ± 1.1
S11	0-5	312 ± 2.1	34.2 ± 1.6	12 ± 1.8	11 ± 1.3
	5-20	174 ± 2.9	21.3 ± 1.0	14 ± 1.5	17 ± 2.1
S12	0-5	626 ± 2.1	19 ± 2.1	27 ± 2.3	29.5 ± 2.0
	5-20	76 ± 3.6	23 ± 1.3	17 ± 1.8	17 ± 1.7
S13	0-5	1564 ± 3.3	27.1 ± 2.0	40 ± 2.0	118 ± 2.1
	5-20	1834 ± 3.4	126 ± 2.3	32 ± 2.6	206 ± 2.5
S14	0-5	518 ± 2.5	11.3 ± 1.6	4.8 ± 1.0	25.2 ± 1.5
	5-20	300 ± 3.1	31 ± 2.1	3.6 ± 1.1	31.9 ± 1.4
S15	0-5	1935 ± 1.5	16 ± 1.6	48 ± 2.1	29.4 ± 1.4
	5-20	1074 ± 2.1	23 ± 1.9	14 ± 1.3	18 ± 1.7
S16	0-5	2718 ± 3.3	40 ± 2.1	46 ± 2.2	78 ± 1.9
	5-20	414 ± 2.4	15.5 ± 1,2	57 ± 2.1	91 ± 2.1
S17	0-5	1064 ± 2.0	64.7 ± 2.3	20 ± 1.3	154 ± 1.9
	5-20	1126 ± 1.6	296 ± 2.6	36 ± 1.1	360 ± 2.3
S18	0-5	38 ± 2.1	30 ± 1.5	26 ± 2.4	54 ± 2.1
	5-20	24 ± 2.6	16 ± 1.0	17 ± 1.8	36 ± 1.7
S19	0-5	376 ± 1.5	28 ± 1.5	19 ± 1.3	16.9 ± 2.6
	5-20	3500 ± 2.4	45 ± 1.1	12.9 ± 1.0	684 ± 3.4
S20	0-5	1170 ± 3.1	39.9 ± 2.0	15.8 ± 1.4	1996 ± 3.7
	5-20	428 ± 2.6	16.4 ± 1.3	12.2 ± 2.0	61 ± 2.1
S21	0-5	50 ± 1.6	16 ± 1.1	24 ± 1.2	65 ± 1.8
	5-20	18 ± 1.3	14 ± 1.0	34 ± 1.1	48 ± 2.1
S22	0-5	558 ± 3.5	12.7 ± 1.3	16.9 ± 2.1	29.7 ± 2.2
	5-20	96 ± 1.8	15.2 ± 1.0	15.4 ± 1.4	31.8 ± 2.7
S23	0-5	1144 ± 2.6	13.9 ± 1.1	56 ± 2.3	236 ± 2.6
	5-20	756 ± 2.0	62.2 ± 1.4	15.1 ± 1.4	88 ± 2.8
S24	0-5	414 ± 2.1	64 ± 1.3	12.3 ± 1.6	16.4 ± 2.1
	5-20	704 ± 1.9	28.4 ± 1.2	28 ± 1.3	37.4 ± 2.0
S25	0-5	42 ± 1.3	19 ± 1.0	18 ± 1.1	15 ± 2.5
	5-20	64 ± 1.0	25 ± 1.1	26 ± 2.1	22 ± 2.3
S26	0-5	26 ± 1.6	1.3 ± 0.9	2.1 ± 1.0	1.2 ± 1.3
	5-20	18.4 ± 2.4	1.6 ± 0.5	2.3 ± 1.0	1.2 ± 0.9

Note: Data are means ± standard deviations.

Table S5. Assessment of soil contamination by heavy metals at depths of 0-5 and 5-20 cm using the contamination factor (C_f), geoaccumulation index (I_{geo}), and potential ecological risk factor (E_r) for sampling sites S1-S26.

Sampling site	Depth, cm	Zn			Cu			Cd			Pb		
		C_f	I_{geo}	E_r	C_f	I_{geo}	E_r	C_f	I_{geo}	E_r	C_f	I_{geo}	E_r
S1	0-5	50.27	13.99	50.27	65.33	6.61	326.67	5.55	3.89	166.50	11.42	3.45	57.08
	5-20	9.41	11.57	9.41	15.53	4.54	77.67	5.75	3.94	172.50	14.67	3.82	73.33
S2	0-5	148.91	15.55	148.91	261.33	8.61	1306.67	19.50	5.70	585.00	68.33	6.04	341.67
	5-20	67.68	14.41	67.68	67.33	6.66	336.67	3.35	3.16	100.50	11.67	3.49	58.33
S3	0-5	26.27	13.05	26.27	14.60	4.45	73.00	3.20	3.09	96.00	16.83	4.01	84.17
	5-20	5.00	10.66	5.00	14.20	4.41	71.00	0.95	1.34	28.50	15.42	3.89	77.08
S4	0-5	200.68	15.98	200.68	784.67	10.20	3923.33	89.50	7.90	2685.00	345.00	8.37	1725.00
	5-20	192.95	15.93	192.95	631.33	9.89	3156.67	80.00	7.74	2400.00	207.50	7.64	1037.50
S5	0-5	148.86	15.55	148.86	137.33	7.69	686.67	31.50	6.39	945.00	69.17	6.05	345.83
	5-20	149.27	15.56	149.27	73.33	6.78	366.67	32.00	6.42	960.00	55.00	5.72	275.00
S6	0-5	164.73	15.70	164.73	218.67	8.36	1093.33	59.00	7.30	1770.00	189.17	7.50	945.83
	5-20	140.27	15.47	140.27	29.40	5.46	147.00	18.00	5.58	540.00	266.67	8.00	1333.33
S7	0-5	147.23	15.54	147.23	86.00	7.01	430.00	44.00	6.87	1320.00	800.00	9.58	4000.00
	5-20	93.64	14.88	93.64	44.60	6.06	223.00	17.50	5.54	525.00	18.25	4.13	91.25
S8	0-5	134.32	15.40	134.32	44.27	6.05	221.33	30.50	6.35	915.00	575.00	9.11	2875.00
	5-20	72.18	14.51	72.18	29.80	5.48	149.00	6.00	4.00	180.00	20.83	4.32	104.17
S9	0-5	99.59	14.97	99.59	146.67	7.78	733.33	30.50	6.35	915.00	280.00	8.07	1400.00
	5-20	73.55	14.53	73.55	15.20	4.51	76.00	16.00	5.42	480.00	19.00	4.19	95.00
S10	0-5	50.77	14.00	50.77	48.00	6.17	240.00	18.05	5.59	541.50	14.08	3.76	70.42
	5-20	17.86	12.49	17.86	22.13	5.05	110.67	14.05	5.23	421.50	29.83	4.84	149.17
S11	0-5	14.18	12.16	14.18	22.80	5.10	114.00	6.00	4.00	180.00	9.17	3.14	45.83
	5-20	7.91	11.32	7.91	14.20	4.41	71.00	7.00	4.22	210.00	14.17	3.77	70.83
S12	0-5	28.45	13.16	28.45	12.67	4.25	63.33	13.50	5.17	405.00	24.58	4.56	122.92
	5-20	3.45	10.12	3.45	15.33	4.52	76.67	8.50	4.50	255.00	14.17	3.77	70.83
S13	0-5	71.09	14.49	71.09	18.07	4.76	90.33	20.00	5.74	600.00	98.33	6.56	491.67
	5-20	83.36	14.72	83.36	84.00	6.98	420.00	16.00	5.42	480.00	171.67	7.36	858.33
S14	0-5	23.55	12.89	23.55	7.53	3.50	37.67	2.40	2.68	72.00	21.00	4.33	105.00
	5-20	13.64	12.10	13.64	20.67	4.95	103.33	1.80	2.26	54.00	26.58	4.67	132.92
S15	0-5	87.95	14.79	87.95	10.67	4.00	53.33	24.00	6.00	720.00	24.50	4.56	122.50
	5-20	48.82	13.94	48.82	15.33	4.52	76.67	7.00	4.22	210.00	15.00	3.85	75.00
S16	0-5	123.55	15.28	123.55	26.67	5.32	133.33	23.00	5.94	690.00	65.00	5.96	325.00
	5-20	18.82	12.57	18.82	10.33	3.95	51.67	28.50	6.25	855.00	75.83	6.19	379.17
S17	0-5	48.36	13.93	48.36	43.13	6.02	215.67	10.00	4.74	300.00	128.33	6.94	641.67
	5-20	51.18	14.01	51.18	197.33	8.21	986.67	18.00	5.58	540.00	300.00	8.17	1500.00
S18	0-5	1.73	9.12	1.73	20.00	4.91	100.00	13.00	5.12	390.00	45.00	5.43	225.00
	5-20	1.09	8.46	1.09	10.67	4.00	53.33	8.50	4.50	255.00	30.00	4.85	150.00
S19	0-5	17.09	12.43	17.09	18.67	4.81	93.33	9.50	4.66	285.00	14.08	3.76	70.42
	5-20	159.09	15.65	159.09	30.00	5.49	150.00	6.45	4.10	193.50	570.00	9.10	2850.00
S20	0-5	53.18	14.07	53.18	26.60	5.32	133.00	7.90	4.40	237.00	1663.33	10.64	8316.67
	5-20	19.45	12.62	19.45	10.93	4.04	54.67	6.10	4.02	183.00	50.83	5.61	254.17
S21	0-5	2.27	9.52	2.27	10.67	4.00	53.33	12.00	5.00	360.00	54.17	5.70	270.83
	5-20	0.82	8.04	0.82	9.33	3.81	46.67	17.00	5.50	510.00	40.00	5.26	200.00
S22	0-5	25.36	13.00	25.36	8.47	3.67	42.33	8.45	4.49	253.50	24.75	4.57	123.75
	5-20	4.36	10.46	4.36	10.13	3.93	50.67	7.70	4.36	231.00	26.50	4.67	132.50
S23	0-5	52.00	14.03	52.00	9.27	3.80	46.33	28.00	6.22	840.00	196.67	7.56	983.33
	5-20	34.36	13.44	34.36	41.47	5.96	207.33	7.55	4.33	226.50	73.33	6.14	366.67
S24	0-5	18.82	12.57	18.82	42.67	6.00	213.33	6.15	4.04	184.50	13.67	3.71	68.33
	5-20	32.00	13.33	32.00	18.93	4.83	94.67	14.00	5.22	420.00	31.17	4.90	155.83
S25	0-5	1.91	9.27	1.91	12.67	4.25	63.33	9.00	4.58	270.00	12.50	3.58	62.50
	5-20	2.91	9.87	2.91	16.67	4.64	83.33	13.00	5.12	390.00	18.75	4.17	93.75
S26	0-5	1.18	8.57	1.18	0.87	0.38	4.33	1.05	1.49	31.50	1.00	-0.06	5.00
	5-20	0.84	8.08	0.84	1.07	0.68	5.33	1.15	1.62	34.50	1.00	-0.06	5.00

Table S6. Ecological importance of plant species at the control site and the industrial area

Family	Species	Important Value	Projective cover (%)	Classification	Control	Industrial site
Sapindaceae	*Acer negundo* L.	0.085	2.35	Perennial	–	+
Salicaceae	*Populus laurifolia* Ledeb.	0.112	3.09	Perennial	+	+
	Populus balsamifera L.	0.094	2.6	Perennial	–	+
	Populus nigra L.	0.088	2.43	Perennial	–	+
	Salix viminalis L.	0.067	1.85	Perennial	–	+
Betulaceae	*Betula pendula* Roth	0.120	3.32	Perennial	+	+
	Betula pubescens Ehrh.	0.073	2.02	Perennial	–	+
Rosaceae	*Padus avium* Mill.	0.071	1.96	Perennial	–	+
Caprifoliaceae	*Lonicera tatarica* L.	0.060	1.66	Perennial	+	–
Pinaceae	*Pinus sylvestris* L.	0.082	2.27	Perennial	–	+
Poaceae	*Calamagrostis epigeios* (L.) Roth	0.135	3.73	Perennial	–	+
	Dactylis glomerata L.	0.128	3.54	Perennial	+	+
	Phleum phleoides (L.) H. Karst.	0.119	3.29	Perennial	+	+
	Poa pratensis L.	0.110	3.04	Perennial	–	+
Asteraceae	*Artemisia vulgaris* L.	0.140	3.87	Perennial	+	+
	Echinops tricholepis Schrenk	0.065	1.8	Perennial	+	–
	Cirsium vulgare (Savi) Ten.	0.090	2.49	Annual or perennial	+	–
	Serratula coronata L.	0.058	1.6	Perennial	+	–
	Tussilago farfara L.	0.097	2.68	Perennial	–	+
	Arctium tomentosum Mill.	0.061	1.69	Annual or perennial	–	+
	Taraxacum officinalis L.	0.064	1.77	Perennial	–	+
	Galatella hauptii (Ledeb.) Lindl. ex DC.	0.055	1.52	Perennial	+	–
	Tripleurospermum perforatum (Merat) M. Lainz	0.060	1.66	Annual	–	+
Malvaceae	*Lavatera thuringiaca* L.	0.058	1.6	Perennial	+	–
Rubiaceae	*Galium boreale* L.	0.061	1.69	Perennial	+	–
	Galium verum L.	0.059	1.63	Perennial	+	–
Geraniaceae	*Geranium pratense* L	0.062	1.71	Perennial	+	–
Rosaceae	*Agrimonia pilosa* Ledeb.	0.060	1.66	Perennial	+	–
	Fragaria viridis Duchesne	0.055	1.52	Perennial	+	–
Brassicaceae	*Bunias orientalis* L.	0.132	3.65	Perennial	+	+
Apiaceae	*Heracleum dissectum* Ledeb.	0.057	1.58	Perennial	+	–

Boraginaceae	*Pulmonaria mollis* Wulf. ex Hornem.	0.056	1.55	Perennial	+	−
Apiaceae	*Bupleurum longifolium* subsp. *aureum* (Fisch. ex Hoffm.) Soo	0.054	1.49	Perennial	+	−
	Conium maculatum L.	0.059	1.63	Annual or perennial	−	+
Fabaceae	*Vicia sepium* L.	0.121	3.34	Perennial	+	+
	Vicia cracca L.	0.104	2.87	Perennial	−	+
Urticaceae	*Urtica dioica* L.	0.118	3.26	Perennial	−	+
Fabaceae	*Trifolium pratense* L.	0.106	2.93	Perennial	−	+
Hypericaceae	*Hypericum perforatum* L.	0.060	1.66	Perennial	+	−
Convolvulaceae	*Convolvulus arvensis* L.	0.125	3.45	Perennial	+	+
Plantaginaceae	*Plantago major* L.	0.063	1.74	Perennial	−	+
Euphorbiaceae	*Euphorbia sp.*	0.057	1.58	Perennial	+	−
Caryophyllaceae	*Gypsophylla altissima* L.	0.056	1.55	Perennial	+	−
Boraginaceae	*Echium vulgare* L.	0.062	1.71	Annual or perennial	−	+

Table S7. Geographic coordinates and environmental characteristics of the soil sampling sites.

Sample	Depth (cm)	Latitude	Longitude	Mesotopography	Parent material	Soil type (WRB)	Vegetation	Functional zone
S1	0–5	50.34868	83.49870	gentle slope	deluvial deposits	Haplic Chernozem	ruderal vegetation	industrial zone
	5–20	50.34868	83.49870	gentle slope	deluvial deposits	Haplic Chernozem	ruderal vegetation	industrial zone
S2	0–5	50.35231	83.50495	gentle slope	deluvial deposits	Haplic Chernozem	ruderal vegetation	industrial zone
	5–20	50.35231	83.50495	gentle slope	deluvial deposits	Haplic Chernozem	ruderal vegetation	industrial zone
S3	0–5	50.34166	83.49445	river terrace	alluvial deposits	Haplic Fluvisol	meadow vegetation	industrial zone
	5–20	50.34166	83.49445	river terrace	alluvial deposits	Haplic Fluvisol	meadow vegetation	industrial zone
S4	0–5	50.34640	83.48613	interfluve	loess-like loams	Haplic Chernozem	steppe vegetation	industrial zone
	5–20	50.34640	83.48613	interfluve	loess-like loams	Haplic Chernozem	steppe vegetation	industrial zone
S5	0–5	50.34654	83.48355	gentle slope	deluvial deposits	Haplic Chernozem	ruderal vegetation	industrial zone
	5–20	50.34654	83.48355	gentle slope	deluvial deposits	Haplic Chernozem	ruderal vegetation	industrial zone
S6	0–5	50.35086	83.48562	river terrace	alluvial deposits	Haplic Fluvisol	meadow vegetation	industrial zone
	5–20	50.35086	83.48562	river terrace	alluvial deposits	Haplic Fluvisol	meadow vegetation	industrial zone
S7	0–5	50.34991	83.48090	slope	deluvial–proluvial deposits	Haplic Chernozem	meadow-steppe vegetation	industrial zone
	5–20	50.34991	83.48090	slope	deluvial–proluvial deposits	Haplic Chernozem	meadow-steppe vegetation	industrial zone
S8	0–5	50.35183	83.48393	river terrace	alluvial deposits	Haplic Fluvisol	meadow vegetation	industrial zone
	5–20	50.35183	83.48393	river terrace	alluvial deposits	Haplic Fluvisol	meadow vegetation	industrial zone
S9	0–5	50.36105	83.47659	interfluve	loess-like loams	Haplic Chernozem	steppe vegetation	industrial zone
	5–20	50.36105	83.47659	interfluve	loess-like loams	Haplic Chernozem	steppe vegetation	industrial zone
S10	0–5	50.35158	83.47304	slope	deluvial deposits	Haplic Chernozem	ruderal vegetation	industrial zone
	5–20	50.35158	83.47304	slope	deluvial deposits	Haplic Chernozem	ruderal vegetation	industrial zone
S11	0–5	50.35798	83.48820	gentle slope	deluvial deposits	Haplic Chernozem	ruderal vegetation	industrial zone
	5–20	50.35798	83.48820	gentle slope	deluvial deposits	Haplic Chernozem	ruderal vegetation	industrial zone
S12	0–5	50.33769	83.49277	river terrace	alluvial deposits	Haplic Fluvisol	meadow vegetation	industrial zone
	5–20	50.33769	83.49277	river terrace	alluvial deposits	Haplic Fluvisol	meadow vegetation	industrial zone
S13	0–5	50.34410	83.50051	interfluve	loess-like loams	Haplic Chernozem	steppe vegetation	agricultural zone
	5–20	50.34410	83.50051	interfluve	loess-like loams	Haplic Chernozem	steppe vegetation	agricultural zone
S14	0–5	50.35228	83.51445	gentle slope	deluvial deposits	Haplic Chernozem	meadow-steppe vegetation	agricultural zone
	5–20	50.35228	83.51445	gentle slope	deluvial deposits	Haplic Chernozem	meadow-steppe vegetation	agricultural zone
S15	0–5	50.36110	83.50329	river terrace	alluvial deposits	Haplic Fluvisol	meadow vegetation	agricultural zone
	5–20	50.36110	83.50329	river terrace	alluvial deposits	Haplic Fluvisol	meadow vegetation	agricultural zone
S16	0–5	50.35786	83.50181	slope	deluvial–proluvial deposits	Haplic Chernozem	meadow-steppe vegetation	agricultural zone
	5–20	50.35786	83.50181	slope	deluvial–proluvial deposits	Haplic Chernozem	meadow-steppe vegetation	agricultural zone

S17	0–5	50.35835	83.52831	interfluve	loess-like loams	Haplic Chernozem	steppe vegetation	agricultural zone
	5–20	50.35835	83.52831	interfluve	loess-like loams	Haplic Chernozem	steppe vegetation	agricultural zone
S18	0–5	50.36325	83.53606	slope	deluvial deposits	Haplic Chernozem	meadow vegetation	agricultural zone
	5–20	50.36325	83.53606	slope	deluvial deposits	Haplic Chernozem	meadow vegetation	agricultural zone
S19	0–5	50.36417	83.54088	slope	deluvial deposits	Haplic Chernozem	meadow vegetation	agricultural zone
	5–20	50.36417	83.54088	slope	deluvial deposits	Haplic Chernozem	meadow vegetation	agricultural zone
S20	0–5	50.36275	83.55311	slope	deluvial deposits	Haplic Chernozem	meadow-steppe vegetation	agricultural zone
	5–20	50.36275	83.55311	slope	deluvial deposits	Haplic Chernozem	meadow-steppe vegetation	agricultural zone
S21	0–5	50.35864	83.55804	interfluve	loess-like loams	Haplic Chernozem	steppe vegetation	agricultural zone
	5–20	50.35864	83.55804	interfluve	loess-like loams	Haplic Chernozem	steppe vegetation	agricultural zone
S22	0–5	50.35331	83.55061	slope	deluvial deposits	Haplic Chernozem	meadow vegetation	agricultural zone
	5–20	50.35331	83.55061	slope	deluvial deposits	Haplic Chernozem	meadow vegetation	agricultural zone
S23	0–5	50.35493	83.53115	river terrace	alluvial deposits	Haplic Fluvisol	meadow vegetation	agricultural zone
	5–20	50.35493	83.53115	river terrace	alluvial deposits	Haplic Fluvisol	meadow vegetation	agricultural zone
S24	0–5	50.34993	83.53469	slope	deluvial–proluvial deposits	Haplic Chernozem	meadow-steppe vegetation	agricultural zone
	5–20	50.34993	83.53469	slope	deluvial–proluvial deposits	Haplic Chernozem	meadow-steppe vegetation	agricultural zone
S25	0–5	50.32602	83.49365	interfluve	loess-like loams	Haplic Chernozem	steppe vegetation	control site
	5–20	50.32602	83.49365	interfluve	loess-like loams	Haplic Chernozem	steppe vegetation	control site
S26	0–5	50.28223	83.32791	river terrace	alluvial deposits	Haplic Fluvisol	meadow vegetation	background/control site
	5–20	50.28223	83.32791	river terrace	alluvial deposits	Haplic Fluvisol	meadow vegetation	background/control site

Table S8. LOD and LOQ values and linear parameters for the HM calibration standards.

HM	R2	Calibration Curve Equation	Range ($mg·L^{-1}$)	LOD ($mg·L^{-1}$)	LOQ ($mg·L^{-1}$)
Pb	0.9996	y = 0.9824x − 0.1931	1.0-10.0	0.05	0.15
Zn	0.9997	y = 1.0128x − 0.0876	1.0-10.0	0.08	0.24
Cd	0.9995	y = 1.1789x − 0.1024	1.0-10.0	0.04	0.12
Cu	0.9996	y = 0.9457x − 0.0642	1.0-10.0	0.07	0.21

Note: R, correlation coefficient; LOD, limit of detection; LOQ, limit of quantification

Table S9. Reference standard certified and measured values and the recovery.

HM	Certified values ($mg·L^{-1}$)	Measured values ($mg·L^{-1}$)	Recovery (%)	Mean recovery ± SD (%)
Pb	5.0	4.98	99.2	99.4 ± 0.32
	10.0	9.95	99.5	
Zn	5.0	4.92	98.4	99.1 ± 1.08
	10.0	9.97	99.7	
Cd	5.0	5.03	100.6	100.3 ± 0.41
	10.0	10.01	100.1	
Cu	5.0	4.98	99.8	100.1 ± 0.36
	10.0	10.04	100.4	

Note: Data are means ± standard deviations

Table S10. Interpretation of the value of the potential environmental risk indicator.

E_r	Levels	R_I	Levels
$E_r < 40$	Low	$R_I < 150$	Low
$40 \le E_r < 80$	Moderate	$150 \le R_I < 300$	Moderate
$80 \le E_r < 160$	Considerable	$300 \le R_I < 600$	Considerable
$160 \le E_r < 320$	High	$R_I \ge 600$	High
$E_r \ge 320$	Very high	$R_I < 150$	Low

Note: E_r is the individual potential ecological risk index of heavy metal; R_I is the comprehensive potential ecological risk index.

Table S11. Classification of Soil Contamination Levels Based on the Geoaccumulation Index (I_{geo}).

Class I_{geo}	Grade I_{geo}	Pollution Grade
Class 0	$I_{geo} \le 0$	uncontaminated
Class 1	$0 < I_{geo} \le 1$	uncontaminated to moderately contaminated
Class 2	$1 < I_{geo} \le 2$	moderately contaminated
Class 3	$2 < I_{geo} \le 3$	moderately to heavily contaminated
Class 4	$3 < I_{geo} \le 4$	heavily contaminated
Class 5	$4 < I_{geo} \le 5$	heavily to extremely contaminated
Class 6	$I_{geo} > 5$	extremely contaminated
