# Supplementary figures and images for: Assessment of Heavy Metal Accumulation in Soils and Dominant Agricultural Crops in an Industrial Environment of Ridder, East Kazakhstan Region
Source: Plants (Basel). 2026 Mar 23;15(6):983. doi: 10.3390/plants15060983 (PMC13030110; doi:10.3390/plants15060983)

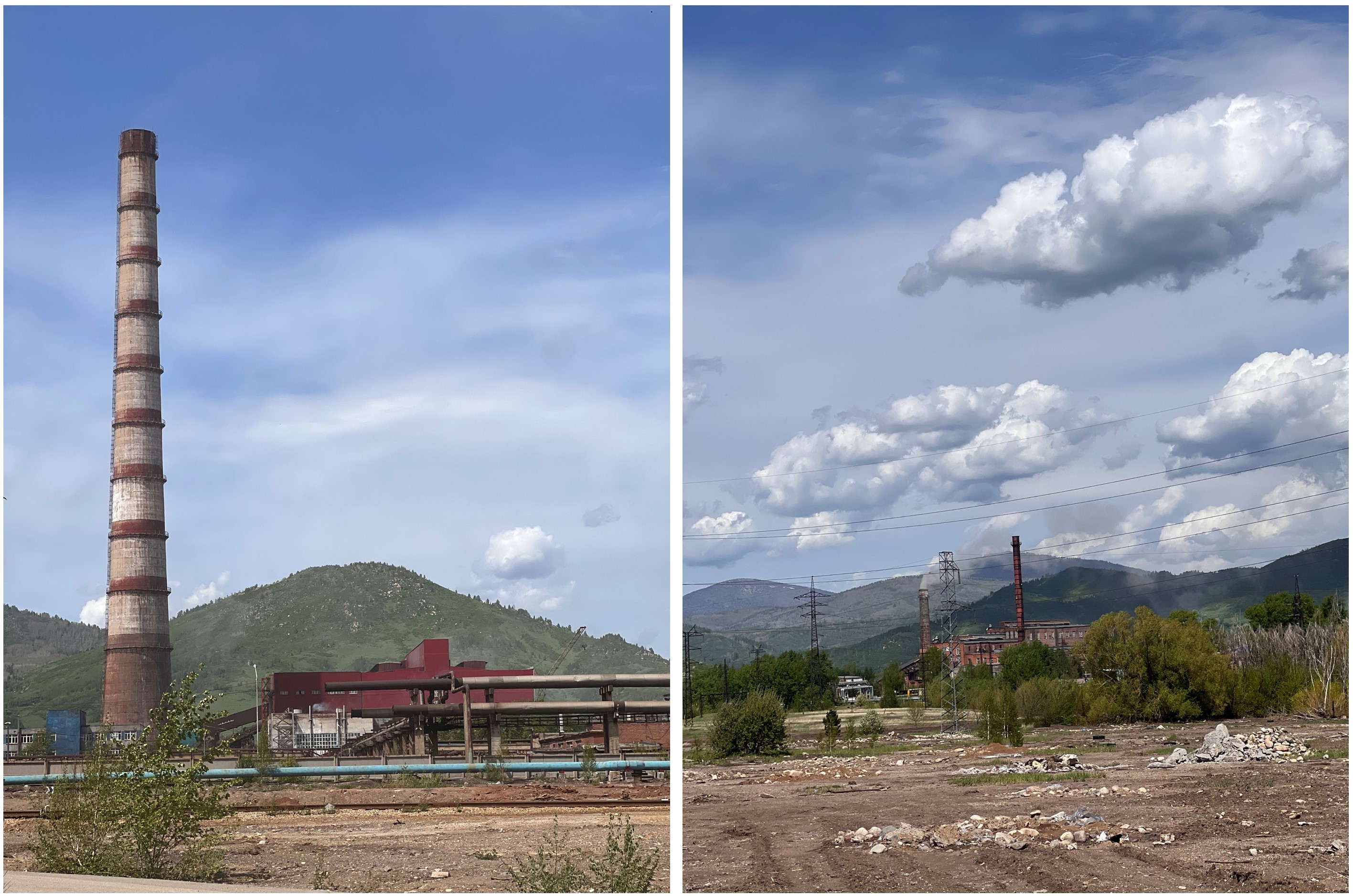

Supplement: Supplementary file 1 [file plants-15-00983-s001.zip › FigureS1_Study area in the Ridder industrial region (East Kazakhstan).jpg]
